# Cost-effectiveness analysis of PD-1 inhibitors combined with chemotherapy as first-line therapy for advanced esophageal squamous-cell carcinoma in China

**DOI:** 10.3389/fphar.2023.1055727

**Published:** 2023-03-02

**Authors:** Shixian Liu, Lei Dou, Shunping Li

**Affiliations:** ^1^ Centre for Health Management and Policy Research, School of Public Health, Cheeloo College of Medicine, Shandong University, Jinan, China; ^2^ NHC Key Laboratory of Health Economics and Policy Research (Shandong University), Jinan, China; ^3^ Center for Health Preference Research, Shandong University, Jinan, China

**Keywords:** cost-effectiveness, esophageal squamous-cell carcinoma, PD-1 inhibitors, first-line therapy, chemoimmunotherapy

## Abstract

**Objective:** This study was aimed to investigate the cost-effectiveness of all available programmed death 1 (PD-1) inhibitors combined with chemotherapy in the first-line treatment of advanced esophageal squamous-cell carcinoma (ESCC) from the Chinese healthcare system perspective.

**Methods:** A partitioned survival model with a 3-week cycle and a 10-year time horizon was constructed based on a network meta-analysis. The survival data and utility values were derived from clinical trials, and the direct medical costs were collected from public drug bidding database and published literature. Total costs, quality-adjusted life-years (QALYs) and incremental cost-effectiveness ratios (ICERs) were calculated. Scenario, one-way and probabilistic sensitivity analyses were performed to assess the uncertainty around model parameters.

**Results:** Compared with mono-chemotherapy, toripalimab, sintilimab and camrelizumab plus chemotherapy were cost-effective treatment regimens, while serplulimab, pembrolizumab and nivolumab plus chemotherapy were not cost-effective options. Toripalimab plus chemotherapy provided the highest QALYs of 0.95 with the lower cost of $8,110.53 compared to other competing alternatives. The robustness of the base-case results was confirmed by scenario and one-way sensitivity analysis. At a willingness-to-pay threshold of three times *per capita* gross domestic product ($38,351.20) in 2021, the probability of toripalimab plus chemotherapy being the optimal option was 74.25% compared with other six competing alternatives.

**Conclusion:** Toripalimab plus chemotherapy represented the most cost-effective option as the first-line therapy for advanced ESCC patients in China.

## Introduction

Esophageal cancer is the fifth most common malignancy and the fourth leading cause of cancer-related death in China (Sung et al., 2021; Zheng et al., 2022). Esophageal squamous cell carcinoma (ESCC) and esophageal adenocarcinoma represent the predominant histological type, with the former accounting for approximately 85% of cases (Arnold et al., 2020). Many esophageal cancers are unresectable at first diagnosis (Rustgi and El-Serag, 2014). Standard fluoropyrimidine or paclitaxel plus cisplatin-based chemotherapy is recommended as first-line treatment for patients with advanced or metastatic ESCC(Muro et al., 2019). The clinical benefits, however, remain limited in patients with advanced or metastatic ESCC receiving standard of care, with a median overall survival (OS) of fewer than 1 year (Ajani et al., 2019; Shah et al., 2023). Therefore, discovering revolutionary treatment strategies to improve prognosis becomes a pressing need in these populations.

In recent years, immune checkpoint inhibitors targeting programmed death 1 (PD-1) or programmed death-ligand 1 (PD-L1) have emerged as promising antitumor regimens across multiple malignancies, including esophageal cancer (Constantinidou et al., 2019). Several prior randomized studies have demonstrated that PD-1 blockade provided significant survival benefits as second-line treatment for advanced ESCC(Kato et al., 2019; Huang et al., 2020). Further, ESCORT-first (Luo et al., 2021), CheckMate-648 (Doki et al., 2022), KEYNOTE-590 (Sun et al., 2021), ORIENT-15 (Lu et al., 2022) and JUPITER-06 (Wang et al., 2022) respectively confirmed that camrelizumab, nivolumab, pembrolizumab, sintilimab and toripalimab combined with chemotherapy produced encouraging antitumor activity compared with mono-chemotherapy. As a result, the five chemoimmunotherapies mentioned above have been in succession approved by the National Medical Products Administration and recommended by the Guidelines of Chinese Society of Clinical Oncology (CSCO, 2022). In 2021, Camrelizumab officially entered the National Reimbursement Drug List (NRDL) negotiation through an 85.2% price reduction for patients with locally advanced or metastatic ESCC, which has progressed after first-line chemotherapy (Cai et al., 2021). The other PD-1 inhibitors covered by the NRDL, such as sintilimab and toripalimab, did not yet include indications related to esophageal cancer.

A published network meta-analysis (NMA) involving five clinical trials with 3,163 patients has investigated the efficacy and safety differences between diverse chemoimmunotherapies in first-line treatment for advanced ESCC (Li et al., 2022). The results proved that toripalimab plus chemotherapy achieved the longest OS [hazard ratio (HR): 0.58, 95% confidence interval (CI): 0.43–0.78], while camrelizumab and sintilimab combined with chemotherapy engendered the longest progression-free survival (PFS) (HR: 0.56, 95% CI: 0.46–0.68) than other treatment examined (Li et al., 2022). Recently, the ASTRUM-007 trial revealed that serplulimab plus chemotherapy significantly improved PFS (HR: 0.60, 95% CI: 0.48–0.75) and OS (HR: 0.68, 95% CI: 0.53–0.87) *versus* mono-chemotherapy for advanced ESCC, but with a manageable safety profile (Song et al., 2023). Considering the lack of head-to-head clinical trials, clinicians confronted insurmountable quandaries in making appropriate treatment options for a given patient based on the available evidence alone, and that is before taking into account relative costs. Therefore, with the enthusiasm of health technology agencies towards life-cycle health technology assessment (Drummond et al., 2008), the selection of optimal treatment options for decision-makers essentially depended on comparative cost-effectiveness (Sanders et al., 2016; Dai et al., 2022).

Most published economic evaluations have assessed the cost-effectiveness of camrelizumab (Zhang et al., 2021), nivolumab (Liu et al., 2022), pembrolizumab (Zhu et al., 2022a) and sintilimab (Ye et al., 2022) compared to chemotherapy in the first-line setting for advanced ESCC. However, the cost-effectiveness between all available first-line chemoimmunotherapies for patients with advanced ESCC was still uncertain. As such, we aimed to evaluate the cost-effectiveness of all first-line chemoimmunotherapies for the treatment of advanced or metastatic ESCC, namely, camrelizumab, nivolumab, pembrolizumab, serplulimab, sintilimab, and toripalimab combined with chemotherapy, and mono-chemotherapy, from the perspective of Chinese healthcare system to better inform reimbursement policy and achieve optimal health resource allocation.

## Methods

### Patients and treatment

This study was guided by the Consolidated Health Economic Evaluation Reporting Standards 2022 (CHEERS 2022) updated reporting guidelines (Supplementary Table S1) (Husereau et al., 2022). This economic evaluation was based on modelling techniques and published literature, and did not require approval of the institutional research ethics board because no real human participants or animals were involved.

A hypothetical cohort of patients, aged at least 18 years, with histologically or cytologically confirmed unresectable locally advanced, recurrent, or metastatic ESCC with the same characteristics as those patients enrolled in ESCORT-first (Luo et al., 2021), CheckMate-648 (Doki et al., 2022), KEYNOTE-590 (Sun et al., 2021), ASTRUM-007 (Song et al., 2023), ORIENT-15 (Lu et al., 2022) and JUPITER-06 (Wang et al., 2022) clinical trials. Eligible patients received one of seven first-line interventions: (1) Chemotherapy (Cisplatin, 75 mg/m^2^, day 1 plus Paclitaxel, 175 mg/m^2^, day 1 or Fluorouracil, 800 mg/m^2^, days 1 through 5; 3-week); (2) Camrelizumab (200 mg; 3-week) plus chemotherapy; (3) Nivolumab (240 mg; 2-week) plus chemotherapy; (4) Pembrolizumab (200 mg; 3-week) plus chemotherapy; (5) Serplulimab (75 mg/kg; 2-week) plus chemotherapy; (6) Sintilimab (200 mg; 3-week) plus chemotherapy; (7) Toripalimab (240 mg; 3-week) plus chemotherapy (Supplementary). After disease progression, we assumed that the remaining patients would receive subsequent best supportive anti-cancer regimens to accurately capture the cost-effectiveness associated with first-line treatment.

## Model construction

**FIGURE 1:**
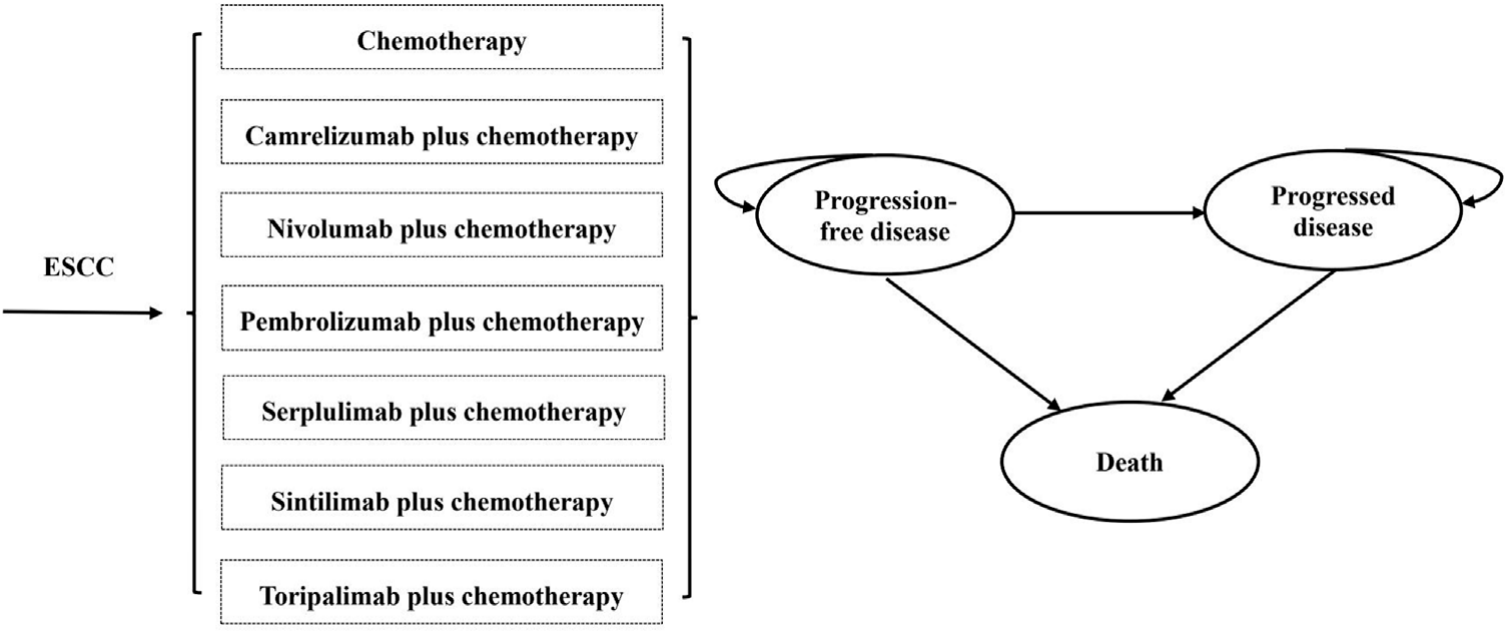
The structure of the partitioned survival model. (ESCC, esophageal squamous-cell carcinoma).

A partitioned survival model was constructed with three exclusive health states [PFS, progression-disease (PD), and death] to portray disease progression and treatment efficacy (Figure 1). The cycle length was 3 weeks, which was consistent with the treatment protocol in clinical trials, and half-cycle correction was implemented to calibrate the timing of events. The 10-year time horizon was adequate to guarantee that ESCC patients completely entered the terminal state. The primary endpoint of the model included overall costs, quality-adjusted life years (QALYs), and incremental cost-effectiveness ratios (ICERs; incremental cost per additional QALY gained) for pairwise comparison between chemoimmunotherapy-related groups. According to China Guidelines for Pharmacoeconomic Evaluations, a discount of 5% was applied to health outcomes and costs beyond the first year over the time horizon (Liu et al., 2020). All costs were adjusted to 2022 prices with the local Consumer Price Index and converted into US dollars (1$ = 6.33 CNY). As recommended by the World Health Organization (Marseille et al., 2015), 3 times *per capita* gross domestic product (GDP) in China in 2021 ($38,351.20) was implemented as the willingness-to-pay (WTP) threshold to investigate the most cost-effective competing alternatives.

### Clinical inputs

As a result of the absence of head-to-head clinical trials comparing chemotherapy and all available chemoimmunotherapies, a systematic review was conducted in February 2023 to identify randomized controlled trials (RCTs) of relevant treatment strategies in advanced ESCC. Web of Science, PubMed, Embase, and Cochrane Library databases were searched using search terms: “camrelizumab or nivolumab or pembrolizumab or serplulimab or sintilimab or toripalimab or PD-1 or PD-L1”, “chemotherapy”, “esophageal squamous cell cancer or esophageal cancer or esophageal carcinoma” and “randomized clinical trial or randomized controlled trial”. The literature search identified 157 publications (Supplementary Figure S1). After rigorous screening, a total of six relevant phase III RCTs with 3,683 patients were included in the systematic review and network meta-analysis. The basic characteristics and bias risk assessment of included studies were summarized in Supplementary Table S2, Figure S2. The results of the network meta-analysis were shown in Supplementary Table S3.

GetData Graph Digitizer 2.26 (http://www.getdata-graph-digitizer.com/) was applied to extract PFS and OS data points from the Kaplan-Meier curves reported in the six RCTs (Supplementary Table S4, S5). To optimally extrapolate the lifetime survival outcome, Guyot’s parametric survival models were considered for each endpoint of chemotherapy (Guyot et al., 2012), including Exponential, Weibull, Log-logistic, Log-normal, and Gompertz distributions (Supplementary Table S6, Figures S3, S4). Weibull distribution provided eligible survival function based on clinical plausibility, statistical goodness-of-fit (Akaike Information Criterion and Bayesian Information Criterion), and visual examination (Latimer, 2013). The estimated shape parameters (*γ*) and scale parameters (*λ*) were shown in Table 1.

**TABLE 1:** Key clinical inputs.

Parameters	Baseline value	Range		Distribution	References
		Minimum	Maximum		
Weibull parameters of PFS and OS for chemotherapy					
ASTRUM 007-PFS	shape: 0.02976800	NA	NA	Weibull	Song et al. (2023)
	scale: 0.45033640				
ASTRUM 007-OS	shape: 0.00768000	NA	NA	Weibull	Song et al. (2023)
	scale: 0.42383320				
CheckMate 648-PFS	shape: 0.0560388	NA	NA	Weibull	Doki et al. (2022)
	scale: 0.1856546				
CheckMate 648-OS	shape: 0.0176300	NA	NA	Weibull	Doki et al. (2022)
	scale: 0.2662113				
ESCORT 1st-PFS	shape: 0.01904830	NA	NA	Weibull	Luo et al. (2021)
	scale: 0.53355410				
ESCORT 1st-OS	shape: 0.00453990	NA	NA	Weibull	Luo et al. (2021)
	scale: 0.54763450				
JUPITER 06-PFS	shape: 0.02086470	NA	NA	Weibull	Wang et al. (2022)
	scale: 0.56311860				
JUPITER 06-OS	shape: 0.00377300	NA	NA	Weibull	Wang et al. (2022)
	scale: 0.60823730				
ORIENT 15-PFS	shape: 0.02568620	NA	NA	Weibull	Lu et al. (2022)
	scale: 0.41099730				
ORIENT 15-OS	shape: 0.00839040	NA	NA	Weibull	Lu et al. (2022)
	scale: 0.42212310				
**HR of PFS in comparison with chemotherapy**					
Camrelizumab plus chemotherapy	0.56	0.46	0.68	Log-normal	NMA
Nivolumab plus chemotherapy	0.81	0.64	1.04	Log-normal	NMA
Pembrolizumab plus chemotherapy	0.65	0.54	0.78	Log-normal	NMA
Serplulimab plus chemotherapy	0.60	0.48	0.75	Log-normal	NMA
Sintilimab plus chemotherapy	0.56	0.46	0.68	Log-normal	NMA
Toripalimab plus chemotherapy	0.58	0.46	0.74	Log-normal	NMA
**HR of OS in comparison with chemotherapy**					
Camrelizumab plus chemotherapy	0.70	0.56	0.88	Log-normal	NMA
Nivolumab plus chemotherapy	0.74	0.58	0.96	Log-normal	NMA
Pembrolizumab plus chemotherapy	0.72	0.60	0.88	Log-normal	NMA
Serplulimab plus chemotherapy	0.68	0.53	0.87	Log-normal	NMA
Sintilimab plus chemotherapy	0.63	0.51	0.78	Log-normal	NMA
Toripalimab plus chemotherapy	0.58	0.43	0.78	Log-normal	NMA
**Risk of severe adverse events (%)**					
**Chemotherapy** ^**#**^					
Anemia	10.61	8.49	12.73	Beta	Average value
Neutropenia	25.36	20.29	30.43	Beta	Average value
Leukopenia	12.58	10.07	15.10	Beta	Average value
Nausea	6.49	5.19	7.78	Beta	Average value
Hypokalemia	6.61	5.29	7.94	Beta	Average value
**Camrelizumab plus chemotherapy**					
Anemia	17.45	13.96	20.94	Beta	Luo et al. (2021)
Leukopenia	24.16	19.33	28.99	Beta	Luo et al. (2021)
Neutropenia	39.93	31.95	47.92	Beta	Luo et al. (2021)
**Nivolumab plus chemotherapy**					
Stomatitis	6.45	5.16	7.74	Beta	Doki et al. (2022)
Anemia	9.68	7.74	11.61	Beta	Doki et al. (2022)
Neutropenia	8.06	6.45	9.68	Beta	Doki et al. (2022)
**Pembrolizumab plus chemotherapy**					
Nausea	7.03	5.62	8.43	Beta	Sun et al. (2021)
Anemia	12.43	9.95	14.92	Beta	Sun et al. (2021)
Fatigue	6.22	4.97	7.46	Beta	Sun et al. (2021)
Neutropenia	22.70	18.16	27.24	Beta	Sun et al. (2021)
Vomiting	6.22	4.97	7.46	Beta	Sun et al. (2021)
Stomatitis	5.68	4.54	6.81	Beta	Sun et al. (2021)
Leukopenia	8.65	6.92	10.38	Beta	Sun et al. (2021)
Hyponatraemia	5.41	4.32	6.49	Beta	Sun et al. (2021)
**Serplulimab plus chemotherapy**					
Anemia	17.54	14.03	21.05	Beta	Song et al. (2023)
Leukopenia	11.26	9.01	13.51	Beta	Song et al. (2023)
Neutropenia	18.59	14.87	22.30	Beta	Song et al. (2023)
**Sintilimab plus chemotherapy**					
Anemia	12.54	10.03	15.05	Beta	Lu et al. (2022)
Leukopenia	17.43	13.94	20.92	Beta	Lu et al. (2022)
Neutropenia	29.97	23.98	35.96	Beta	Lu et al. (2022)
**Toripalimab plus chemotherapy**					
Anemia	10.89	8.72	13.07	Beta	Wang et al. (2022)
Leukopenia	20.23	16.19	24.28	Beta	Wang et al. (2022)
Neutropenia	42.41	33.93	50.89	Beta	Wang et al. (2022)
Pneumonia	5.84	4.67	7.00	Beta	Wang et al. (2022)

#, The incidence of adverse events associated with the chemotherapy group was derived from the mean of ESCORT-first, CheckMate-648, KEYNOTE-590, ASTRUM-007, ORIENT-15, and JUPITER-06, clinical trials; PFS, progression-free survival; OS, overall survival; HR, hazard ratio; NMA, network meta-analysis.

The baseline hazards for chemotherapy were estimated by averaging the patient survival data fitted by Weibull distribution (Supplementary Figure S5). We then derived the expected survival curves for chemoimmunotherapies by applying the HRs to the reference arm of chemotherapy. The Weibull parameter γ for chemoimmunotherapies was equal to the reference arm, and the Weibull parameter λ for chemoimmunotherapies was calculated as λ for reference arm multiplied by the HRs between alternative treatments and mono-chemotherapy (Hoyle et al., 2010).

### Cost inputs

Our model considered only direct medical costs, which included drug costs, subsequent treatment, hospitalization expense, routine follow-up and radiological examinations, and administration costs associated with adverse events (AEs) (Table 2). To estimate drug costs, we calculated the average winning bids in 2023 from YAOZHI database (https://data.yaozh.com/), which aggregated the latest price data around the country. The default height of 165 cm and body weight of 65 kg, with an average body surface area (BSA) of 1.72 m^2^ were assumed for the Chinese ESCC patients to determine the dosage and expenditure of chemotherapies (Liu et al., 2022). Other healthcare-related costs were retrieved from recently published literature (Liu et al., 2022; Shen et al., 2022). Grade 3 or above AEs with an incidence of greater than 5% reported in the clinical trial were included as they exerted a considerable effect on the course of survival and treatment, including anemia, neutropenia, leukopenia, stomatitis, nausea, fatigue, vomiting, hyponatraemia, hypokalemia and pneumonia (Liu et al., 2022; Shao et al., 2022; Zhan et al., 2022). For each treatment regimen, the management cost of serious AEs were determined by multiplying the unite cost (per event) by the corresponding incidence rate.

**TABLE 2:** Basic parameters input to the model and the ranges of the sensitivity analyses.

Parameters	Baseline value	Range		Distribution	References
		Minimum	Maximum		
Cost inputs (US $)					
Camrelizumab (200 mg)	462.25	369.80	554.69	Gamma	YaoZH (2023)
Nivolumab (100 mg)	1460.30	1168.24	1752.36	Gamma	YaoZH (2023)
Pembrolizumab (100 mg)	2828.73	2262.98	3394.47	Gamma	YaoZH (2023)
Serplulimab (100 mg)	882.18	705.74	1058.62	Gamma	YaoZH (2023)
Sintilimab (100 mg)	170.50	136.40	204.60	Gamma	YaoZH (2023)
Toripalimab (240 mg)	302.00	241.60	362.40	Gamma	YaoZH (2023)
Cisplatin (10 mg)	1.47	1.18	1.77	Gamma	YaoZH (2023)
Paclitaxel (30 mg)	10.61	8.49	12.73	Gamma	YaoZH (2023)
Fluorouracil (250 mg)	8.51	6.81	10.22	Gamma	YaoZH (2023)
Cost of best supportive care	182.23	145.78	218.68	Gamma	Liu et al. (2022)
Hospitalization expense	19.86	15.89	12.83	Gamma	Shen et al. (2022)
Routine follow-up cost	73.72	58.98	88.47	Gamma	Liu et al. (2022)
Cost of laboratory tests and radiological examinations	357.34	285.87	428.81	Gamma	Liu et al. (2022)
Management cost of Anemia	336.63	269.30	403.95	Gamma	Zhan et al. (2022)
Management cost of Neutropenia	454.26	363.41	545.11	Gamma	Liu et al. (2022)
Management cost of Leukopenia	454.26	363.41	545.11	Gamma	Liu et al. (2022)
Management cost of Stomatitis	46.54	37.23	55.85	Gamma	Liu et al. (2022)
Management cost of Nausea	101.15	80.92	121.38	Gamma	Zhan et al. (2022)
Management cost of Fatigue	113.59	90.87	136.31	Gamma	Liu et al. (2022)
Management cost of Vomiting	101.15	80.92	121.38	Gamma	Zhan et al. (2022)
Management cost of Hyponatraemia	3223.00	2578.40	3867.60	Gamma	Shao et al. (2022)
Management cost of Pneumonia	1640.00	1312.00	1968.00	Gamma	Shao et al. (2022)
Management cost of Hypokalemia	3000.00	2400.00	3600.00	Gamma	Assumption
**Utility inputs**					
Utility of PFS	0.75	0.60	0.90	Beta	Wilke et al. (2014)
Utility of progression-disease	0.60	0.48	0.72	Beta	Wilke et al. (2014)
Disutility of Anemia	0.07	0.06	0.09	Beta	Cai et al. (2021)
Disutility of Neutropenia	0.20	0.16	0.24	Beta	Nafees et al. (2017)
Disutility of Leukopenia	0.20	0.16	0.24	Beta	Nafees et al. (2017)
Disutility of Stomatitis	0.15	0.12	0.18	Beta	Lloyd et al. (2006)
Disutility of Nausea	0.13	0.10	0.15	Beta	Nafees et al. (2017)
Disutility of Fatigue	0.07	0.05	0.08	Beta	Nafees et al. (2017)
Disutility of Vomiting	0.13	0.10	0.15	Beta	Nafees et al. (2017)
Disutility of Hyponatraemia	0.03	0.02	0.04	Beta	Shao et al. (2022)
Disutility of Pneumonia	0.05	0.04	0.06	Beta	Shao et al. (2022)
Disutility of Hypokalemia	0.03	0.02	0.04	Beta	Assumption
**Others**					
Discount rate (%)	5.00	0.00	8.00	Beta	Liu et al. (2020)
Patient weight (kg)	65.00	52.00	78.00	Gamma	Liu et al. (2022)
Body surface area (m^2^)	1.72	1.38	2.06	Gamma	Liu et al. (2022)

### Health state utility

Health state utilities were estimated based on the EuroQoL five-dimension, three-level questionnaire reported from a double-blind, randomised phase 3 trial, which recruited participants with metastatic or locally advanced gastric or gastro-oesophageal junction adenocarcinoma (Wilke et al., 2014). The baseline utility values for PFS and PD states were 0.75 and 0.60, respectively, which were in compliance with previously published cost-effectiveness analyses (Yang et al., 2021; Liu et al., 2022). The disutility values caused by grade 3 or above treatment-related AEs were considered by multiplying the duration-adjusted disutilities by the prevalence rates of specific AEs (Lloyd et al., 2006; Nafees et al., 2017; Cai et al., 2021; Shao et al., 2022) (Table 2).

### Scenario and sensitivity analyses

We performed four scenarios to examine how our model was impacted by time horizon, utility values, BSA and subsequent treatment strategies: first, health utility values from published economic evaluations associated with ESCC were employed to further validate the base-case results (Zhang et al., 2020; Marguet et al., 2021; Zhang et al., 2021); second, shorter time horizon (2, 5, and 8 years) was conducted in this scenario; third, the reasonably lower or higher weight and BSA (58 kg, 1.60 m^2^ and 80 kg, 1.98 m^2^) were investigated; fourth, according to guidelines and clinical trials (CSCO, 2022), after disease progression, we assumed that the proportion of patients receiving immunotherapy, targeted therapy, chemotherapy and BSC in the chemotherapy and chemoimmunotherapy groups were 10% and 20%, 10% and 10%, 20% and 25%, and 60% and 45%, respectively.

One-way and probabilistic sensitivity analyses (PSA) were conducted for input parameters to explore the robustness of our results. In the one-way sensitivity analyses, the estimated range of variables were either based on reported 95% confidence intervals or determined by assuming a 20% deviation from the base-case values to appraise their degree of impact on ICERs. On the basis of China Guidelines for Pharmacoeconomic Evaluations, the range of discount rate was set as 0%–8% (Liu et al., 2020). The results were represented by Tornado diagrams. For the PSA, 10,000 Monte Carlo simulations was generated by simultaneously sampling all crucial variables from the pre-specified statistical distributions. Gamma distribution was selected for costs, log-normal distribution for HRs between the competing alternatives, and beta distribution for utility values and proportions (Briggs et al., 2012). The results of PSA were presented in cost-effectiveness acceptability curves (CEAC), which illustrated the probabilities of each competing strategy being cost-effective at various WTP thresholds.

## Results

### Base-case results

The base-case results were shown in Table 3. Compared with mono-chemotherapy, the ICERs of toripalimab, sintilimab, and camrelizumab combined with chemotherapy were $14,047.53/QALY, $18,622.34/QALY, and $29,771.17/QALY, respectively, all were lower than WTP threshold. The ICERs of serplulimab, pembrolizumab, and nivolumab plus chemotherapy *versus* mono-chemotherapy were $170,911.36/QALY, $211,350.41/QALY, and $400,768.95/QALY, respectively, all were more than WTP threshold. In the pairwise comparison between all competing treatments, toripalimab plus chemotherapy yielded the highest QALYs (0.95) with lower cost ($8,110.53) and represented high-value option for advanced ESCC patients at the current price and WTP threshold.

**TABLE 3:** Base-case results.

Strategy	Total cost	QALYs	ICER ($/QALY, pairwise comparison)					
Chemotherapy	4,436.40	0.69	-	-	-	-	-	-
Toripalimab plus chemotherapy	8,110.53	0.95	14,047.53	-	-	-	-	-
Sintilimab plus chemotherapy	8,643.48	0.91	18,622.34	dominated	-	-	-	-
Camrelizumab plus chemotherapy	9,656.62	0.86	29,771.17	dominated	dominated	-	-	-
Serplulimab plus chemotherapy	36,370.68	0.87	170,911.36	dominated	dominated	2,322,505.88	-	-
Pembrolizumab plus chemotherapy	37,312.48	0.84	211,350.41	dominated	dominated	dominated	dominated	-
Nivolumab plus chemotherapy	56,972.21	0.82	400,768.95	dominated	dominated	dominated	dominated	dominated

QALYs, quality-adjusted life years; ICER, incremental cost-effectiveness ratios.

### Scenario and sensitivity analyses results

**FIGURE 2:**
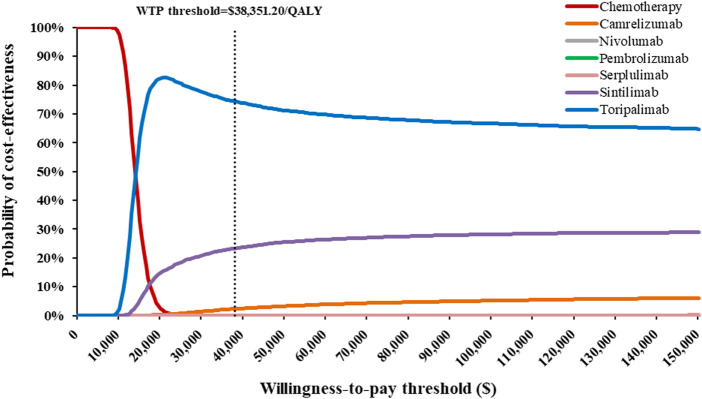
Cost-effectiveness acceptability curves indicating the probability of each treatment regimen to be cost-effectiveness in the treatment of advanced esophageal squamous-cell carcinoma at various willingness-to-pay thresholds in China.

Across all scenario analyses, the general conclusions of the primary analyses were robust and reliable, namely, toripalimab plus chemotherapy was the most cost-effective option against competing regimens (Supplementary Tables S7, S8, S9, S10). One-way sensitivity analyses demonstrated that HR-related parameters, drug costs, utility values and BSA played a considerable role in the base-case results, but alterations in these variables did not significantly alter the conclusion (Supplementary Figure S6). At the WTP thresholds of 3 times *per capita* GDP in China, the CEAC revealed that approximately 74.25%, 23.38%, and 2.37% probabilities of toripalimab, sintilimab, and camrelizumabplus chemotherapy being cost-effective options in simultaneous comparisons of competing strategies (Figure 2).

## Discussion

To our knowledge, this is the first study to comprehensively appraise the cost-effectiveness of currently available first-line chemoimmunotherapies for patients with advanced ESCC from the Chinese healthcare system perspective. Our findings indicated that toripalimab, sintilimab, and camrelizumab combined with chemotherapy were cost-effectiveness compared to chemotherapy. Toripalimab plus chemotherapy was the most cost-effective treatment paradigm under the current WTP threshold by virtue of the highest QALYs and lower cost. The base-case results were upheld by the scenario and sensitivity analyses.

Toripalimab was the first approved PD-1 inhibitor developed independently by Chinese pharmaceutical companies, which not only greatly reduced transportation costs compared to imported immunotherapeutic agents, but also provided more substantial price reductions than comparable inhibitors (Tian et al., 2022). Therefore, toripalimab could be more accessible and widely applied for Chinese patients. The NMA demonstrated that sintilimab and camrelizumab plus chemotherapy provided more significant improvements in PFS and OS than nivolumab and pembrolizumab plus chemotherapy. Due to the considerable price advantage and accessibility, sintilimab and camrelizumab plus chemotherapy may be appropriate alternatives for advanced ESCC patients. Serplulimab, a novel domestic PD-1 inhibitor, plus chemotherapy for first-line treatment has not shown an economic advantage, although it may be cost-effective in patients with extensive-stage small cell lung cancer (Zhu et al., 2022b). Therefore, a substantial price reduction for serplulimab was essential to improve patient affordability. Moreover, PD-1 inhibitors plus chemotherapy improved clinical benefits as first-line therapy for advanced ESCC patients, at the cost of greater but controllable toxicity including increased frequency of serious AEs (Li et al., 2022). However, one-way sensitivity analyses showed that these tolerable toxicity-related costs and disutilities exerted a minimal impact on cost-effectiveness and, hence, would not substantially alter the results.

In recent years, the Chinese self-developed innovative PD-1 inhibitors have gradually provided better survival benefits, clinical tolerability and cost-effective treatment options for various cancer patients. This situation is mainly driven by the centralized price-negotiated mechanisms to improve the accessibility and afordability of patients (Zhang et al., 2022a; Zhang et al., 2022b). The National Medical Products Administration, previously called the China Food and Drug Administration, has strengthened regulatory capacity and launched a series of priority procedures to expedite the development, review and approval of innovative anti-cancer medicines (Zhou et al., 2017; Zhang et al., 2022a). Furthermore, to temper rapidly increasing costs, value-based pricing and national medical insurance negotiations became critical criterion for innovative drugs to be covered by national medical insurance (Si et al., 2020; Tang et al., 2020). These mechanisms have reduced drug prices by half, safeguarding both patient affordability and the sustainability of medical insurance (Zhang et al., 2022b).

To date, several economic evaluations were relevant to ours and warrant discussion. Zhang et al. (Zhang et al., 2021) estimated the cost-effectiveness of camrelizumab plus chemotherapy in the first-line treatment of advanced or metastatic ESCC based on ESCORT-first clinical trial, and suggested that camrelizumab plus chemotherapy might not be cost-effective compared with standard chemotherapy in China. Nevertheless, this previous assessment used non-negotiated prices for camrelizumab, which are no longer relevant at present, as the medical insurance negotiation mechanism has dramatically improved accessibility for patients. Zhu et al. (Zhu et al., 2022a) and Liu et al. (Liu et al., 2022) evaluated the cost-effectiveness of pembrolizumab and nivolumab combined with chemotherapy from the Chinese healthcare system perspective, respectively, and the conclusions aligned well with those of this analysis. Nivolumab and pembrolizumab combined with chemotherapy was extremely unlikely to be economical compared to chemotherapy (Malmberg et al., 2022), and substantial price reductions or generous patient assistance programs were required to improve affordability (Howard, 2014). The latest economic evidence suggested that sintilimab and toripalimab plus chemotherapy were cost-effective compared with chemotherapy regimens in the first-line treatment of patients with advanced ESCC(Shao et al., 2022; Fang et al., 2023). Our results were consistent with available studies. Camrelizumab, sintilimab, and toripalimab plus chemotherapy were high-value innovative options for advanced ESCC patients in China.

Our study had some limitations that merited discussion, many of which were governed by data availability and model assumptions. Foremost, because the head-to-head clinical trial was unavailable, an indirect comparison was performed based on NMA to evaluate all available chemoimmunotherapies as first-line treatment for advanced ESCC, although there was moderate heterogeneity in the pairwise comparison. Second, we assumed best supportive care as the primary treatment after disease progression, which might be different from the actual clinical situations. Scenario analysis demonstrated that the alternative of subsequent treatment options would not substantially alter the outcome of the base-case analysis. Third, since the utility values of specific health states were limited in China, the utilities and disutilities were determined based on published clinical trial, which might cause some deviations in the cumulative QALYs. Fourth, due to the absence of data, the costs and disutilities associated with grade 1/2 treatment-related AEs were excluded from this model, although one-way sensitivity analyses implied that only minimal impact on the base-case results. Fifth, PD-L1 expression was enriched in ESCC patients. Prior economic evidence indicated that PD-1 inhibitors were potentially more sensitive to PD-L1-positive ESCC patients against overall population (Zhu et al., 2022a; Liu et al., 2022; Shao et al., 2022). Because PD-L1-positive was inconsistently defined across clinical trials, subgroup analyses were not feasible in this study. Consequently, subgroup analyses based on head-to-head trials or real-world data warranted further studies to support healthcare decision-making and precision medicine.

## Conclusion

In summary, our findings showed that toripalimab, sintilimab, and camrelizumab combined with chemotherapy were cost-effective treatment options over chemotherapy, and toripalimab plus chemotherapy was the most cost-effective regimen compared with other competing alternatives as the first-line treatment for advanced ESCC patients in China.

## Data Availability

The original contributions presented in the study are included in the article/Supplementary Material, further inquiries can be directed to the corresponding author.
