# Supplementary material for: Cost-effectiveness analysis of PD-1 inhibitors combined with chemotherapy as first-line therapy for advanced esophageal squamous-cell carcinoma in China
Source: Front Pharmacol. 2023 Mar 2;14:1055727. doi: 10.3389/fphar.2023.1055727 (PMC10017726; doi:10.3389/fphar.2023.1055727)
Supplement: Supplementary file 1 [file DataSheet1.PDF]

Cost-effectiveness Analysis of PD-1 inhibitors combined with chemotherapy as first-line therapy for advanced esophageal squamous-cell carcinoma in China

Shixian Liu[1,2,3], Lei Dou[1,2,3], and Shunping Li[1,2,3*]

[1]Centre for Health Management and Policy Research, School of Public Health, Cheeloo College of Medicine, Shandong University, Jinan, China,

[2]NHC Key Laboratory of Health Economics and Policy Research (Shandong University), Jinan, China,

[3]Center for Health Preference Research, Shandong University, Jinan, China

[*]**Correspondence:**

Shunping Li

lishunping@sdu.edu.cn

Treatment Strategies

(1) Chemotherapy: Cisplatin (75 mg/m^2 on day 1 every 3 weeks) plus paclitaxel (175 mg/m^2 on day 1 every 3 weeks) or fluorouracil (800 mg/m^2 on days 1-5 every 3 weeks).

(2) Camrelizumab plus chemotherapy：Camrelizumab (200 mg on day 1 every 3 weeks), plus Paclitaxel (175 mg/m^2 on day 1 every 3 weeks) and cisplatin (75 mg/m^2 on day 1 every 3 weeks) for up to 6 cycles after randomization.

(3) Nivolumab plus chemotherapy: Nivolumab (240 mg on day 1 every 2 weeks), plus Cisplatin (80 mg/m^2 on day 1 every 4 weeks) and fluorouracil (800 mg/m^2 on days 1-5 every 4 weeks) for up to 6 cycles after randomization.

(4) Pembrolizumab plus chemotherapy: Pembrolizumab (200 mg on day 1 every 3 weeks), plus Cisplatin (80 mg/m^2 on day 1 every 3 weeks) and fluorouracil (800 mg/m^2 on days 1-5 every 3 weeks) for up to 6 cycles after randomization.

(5) Serplulimab plus chemotherapy: Serplulimab (3 mg/kg on day 1 every 2 weeks), plus Cisplatin (50 mg/m^2 on day 1 every 2 weeks for up to 8 cycles) and fluorouracil (1,200 mg/m^2 on days 1-2 every 2 weeks for up to 12 cycles).

(6) Sintilimab plus chemotherapy: Sintilimab (3 mg/kg in patients weighing <60 kg or 200 mg in patients weighing ≥60 kg on day 1 every 3 weeks), Cisplatin (75 mg/m2 on day 1 every 3 weeks) plus Paclitaxel (87.5 mg/m^2 on day 1 and day 8 of cycle 1; 175 mg/m^2 on day 1 of the other cycles) or Fluorouracil (800 mg/m^2 on days 1-5 every 3 weeks) for up to 6 cycles after randomization.

(7) Toripalimab plus chemotherapy: Toripalimab (240 mg on day 1 on day 1), plus Paclitaxel (175 mg/m^2 on day 1 every 3 weeks) and cisplatin (75 mg/m2 on day 1 every 3 weeks) for up to 6 cycles after randomization.

Supplementary Materials

Supplementary Table 1 | CHEERS Checklist 2022.

Supplementary Figure 1 | Flow diagram of literature retrieval and selection.

Supplementary Table 2 | Basic characteristics of the included studies.

Supplementary Figure 2 | Risk of bias assessment results.

Supplementary Table 3 | Results of the network meta-analysis.

Supplementary Table 4 | Individual patient data for chemotherapy (PFS).

Supplementary Table 5 | Individual patient data for chemotherapy (OS).

Supplementary Table 6 | Summary of statistical goodness-of-fit of Kaplan-Meier curves for chemotherapy.

Supplementary Figure 3 | The reconstructed Kaplan-Meier PFS curves of chemotherapy.

Supplementary Figure 4 | The reconstructed Kaplan-Meier OS curves of chemotherapy.

Supplementary Figure 5 | PFS and OS curves for the chemotherapy group fitted by Weibull distribution.

Supplementary Table 7 | Scenario analysis results for different sources of utility values.

Supplementary Table 8 | Scenario analysis results of various time horizons.

Supplementary Table 9 | Scenario analysis results related to lower and higher patient weight and body surface area.

Supplementary Table 10 | Scenario analysis results related to subsequent treatment.

Supplementary Figure 6 | Tornado diagrams of one-way sensitivity analyses.

Supplementary Table 1 | CHEERS Checklist 2022.

Section	Item No	Guidance for reporting	Reported
**Title**			
Title	1	Identify the study as an economic evaluation and specify the interventions being compared	Yes
**Abstract**			
Abstract	2	Provide a structured summary that highlights context, key methods, results, and alternative analyses	Yes
**Introduction**			
Background and objectives	3	Give the context for the study, the study question, and its practical relevance for decision making in policy or practice	Yes
**Methods**			
Health economic analysis plan	4	Indicate whether a health economic analysis plan was developed and where available	Yes
Study population	5	Describe characteristics of the study population (such as age range, demographics, socioeconomic, or clinical characteristics)	Yes
Setting and location	6	Provide relevant contextual information that may influence findings	Yes
Comparators	7	Describe the interventions or strategies being compared and why chosen	Yes
Perspective	8	State the perspective(s) adopted by the study and why chosen	Yes
Time horizon	9	State the time horizon for the study and why appropriate	Yes
Discount rate	10	Report the discount rate(s) and reason chosen	Yes
Selection of outcomes	11	Describe what outcomes were used as the measure(s) of benefit(s) and harm(s)	Yes
Measurement of outcomes	12	Describe how outcomes used to capture benefit(s) and harm(s) were measured	Yes
Valuation of outcomes	13	Describe the population and methods used to measure and value outcomes	Yes
Measurement and valuation of resources and costs	14	Describe how costs were valued	Yes
Currency, price date, and conversion	15	Report the dates of the estimated resource quantities and unit costs, plus the currency and year of conversion	Yes
Rationale and description of model	16	If modelling is used, describe in detail and why used. Report if the model is publicly available and where it can be accessed	Yes
Analytics and assumptions	17	Describe any methods for analysing or statistically transforming data, any extrapolation methods, and approaches for validating	Yes

		any model used	
Characterising heterogeneity	18	Describe any methods used for estimating how the results of the study vary for subgroups	Yes
Characterising distributional effects	19	Describe how impacts are distributed across different individuals or adjustments made to reflect priority populations	Yes
Characterising uncertainty	20	Describe methods to characterise any sources of uncertainty in the analysis	Yes
Approach to engagement with patients and others affected by the study	21	Describe any approaches to engage patients or service recipients, the general public, communities, or stakeholders (such as clinicians or payers) in the design of the study	Yes
**Results**			
Study parameters	22	Report all analytic inputs (such as values, ranges, references) including uncertainty or distributional assumptions	Yes
Summary of main results	23	Report the mean values for the main categories of costs and outcomes of interest and summarise them in the most appropriate overall measure	Yes
Effect of uncertainty	24	Describe how uncertainty about analytic judgments, inputs, or projections affect findings. Report the effect of choice of discount rate and time horizon, if applicable	Yes
Effect of engagement with patients and others affected by the study	25	Report on any difference patient/service recipient, general public, community, or stakeholder involvement made to the approach or findings of the study	Yes
**Discussion**			
Study findings, limitations, generalisability, and current knowledge	26	Report key findings, limitations, ethical or equity considerations not captured, and how these could affect patients, policy, or practice	Yes
Other relevant information Source of funding	27	Describe how the study was funded and any role of the funder in the identification, design, conduct, and reporting of the analysis	Yes
Conflicts of interest	28	Report authors conflicts of interest according to journal or International Committee of Medical Journal Editors requirements	Yes

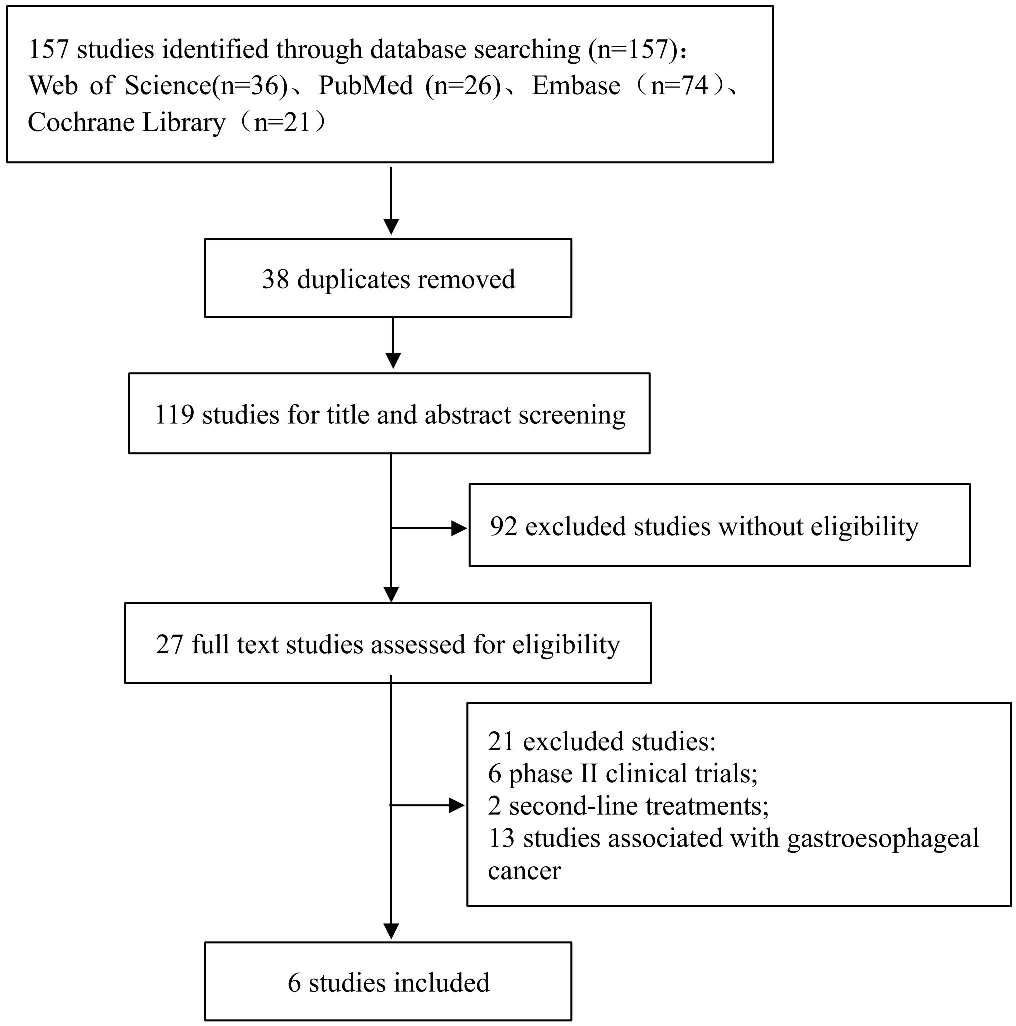

Supplementary Figure 1 | Flow diagram of literature retrieval and selection.

Supplementary Table 2 | Basic characteristics of the included studies.

Study	Experiment				Control				HR for PFS	HR for OS
	Treatment	Patients	Median age (range)	Male (%)	Treatment	Patients	Median age (range)	Male (%)		
ASTRUM-007	Serplulimab+ chemotherapy	368	64	317 (86)	Chemotherapy	183	64	153 (84)	0.60 (0.48-0.75)	0.68 (0.53-0.87)
CheckMate-648	Nivolumab+ chemotherapy	310	64 (40–90)	253 (79)	Chemotherapy	304	64 (26–81)	275 (85)	0.81 (0.64-1.04)	0.74 (0.58-0.96)
ESCORT-1st	Camrelizumab+ chemotherapy	298	62	260 (87)	Chemotherapy	298	62	263 (88)	0.56 (0.46-0.68)	0.70 (0.56-0.88)
JUPITER-06	Toripalimab+ chemotherapy	257	63 (20–75)	217 (84)	Chemotherapy	257	62 (40–74)	220 (86)	0.58 (0.46-0.74)	0.58 (0.43-0.78)
KEYNOTE-590	Pembrolizumab+ chemotherapy	373	64 (28–94)	306 (82)	Chemotherapy	376	62 (27–89)	319 (85)	0.65 (0.54-0.78)	0.72 (0.60-0.88)
ORIENT-15	Sintilimab+ chemotherapy	327	63	279 (85)	Chemotherapy	332	63	288 (87)	0.56 (0.46-0.68)	0.63 (0.51-0.78)

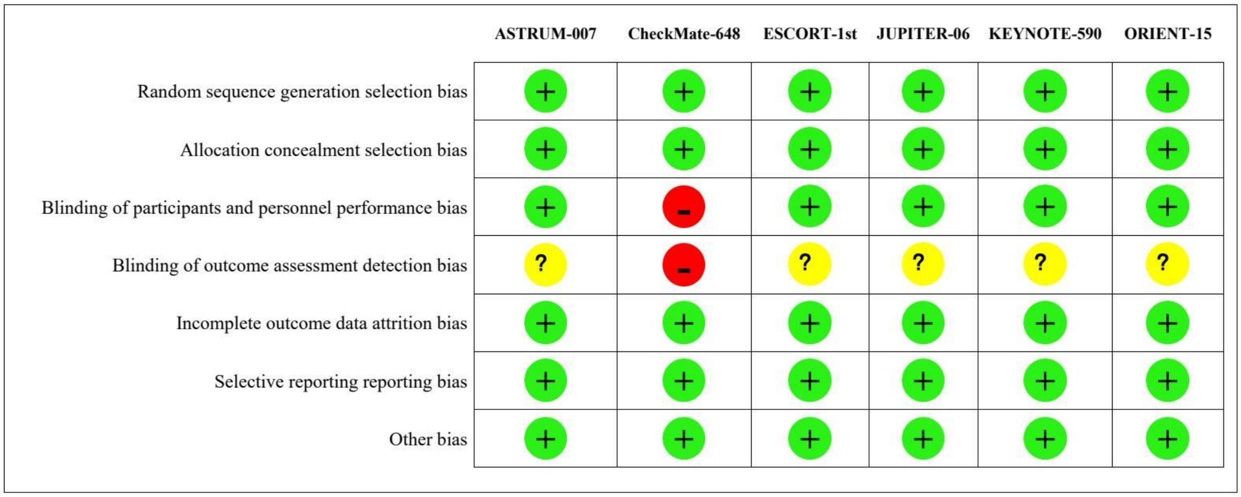

Supplementary Figure 2 | Risk of bias assessment results.

Supplementary Table 3 | Results of the network meta-analysis. (A: PFS; B: OS)

A. PFS

**Chemotherapy**	**0.60 (0.48, 0.75)**	0.81 (0.64, 1.03)	**0.56 (0.46, 0.68)**	**0.58 (0.46, 0.74)**	**0.65 (0.54, 0.78)**	**0.56 (0.46, 0.68)**
1.67 (1.34, 2.09)	**Serplulimab**	1.35 (0.97, 1.88)	0.93 (0.69, 1.25)	0.97 (0.70, 1.34)	1.08 (0.81, 1.45)	0.93 (0.69, 1.26)
1.24 (0.97, 1.57)	0.74 (0.53, 1.03)	**Nivolumab**	**0.69 (0.51, 0.95)**	0.72 (0.51, 1.01)	0.80 (0.59, 1.09)	**0.69 (0.51, 0.94)**
1.79 (1.47, 2.17)	1.07 (0.80, 1.44)	1.45 (1.06, 1.97)	**Camrelizumab**	1.04 (0.76, 1.40)	1.16 (0.89, 1.52)	1.00 (0.76, 1.32)
1.72 (1.36, 2.19)	1.04 (0.75, 1.43)	1.40 (0.99, 1.96)	0.97 (0.71, 1.31)	**Toripalimab**	1.12 (0.83, 1.52)	0.97 (0.71, 1.31)
1.54 (1.28, 1.85)	0.92 (0.69, 1.23)	1.25 (0.92, 1.69)	0.86 (0.66, 1.12)	0.89 (0.66, 1.20)	**Pembrolizumab**	0.86 (0.66, 1.13)
1.79 (1.47, 2.17)	1.07 (0.80, 1.44)	1.45 (1.06, 1.98)	1.00 (0.76, 1.32)	1.04 (0.76, 1.42)	1.16 (0.89, 1.52)	**Sintilimab**

B. OS

**Chemotherapy**	**0.68 (0.53, 0.87)**	**0.74 (0.58, 0.95)**	**0.70 (0.56, 0.88)**	**0.58 (0.43, 0.78)**	**0.72 (0.59, 0.87)**	**0.63 (0.51, 0.78)**
1.47 (1.15, 1.88)	**Serplulimab**	1.09 (0.77, 1.55)	1.03 (0.74, 1.45)	0.85 (0.58, 1.26)	1.06 (0.78, 1.45)	0.93 (0.67, 1.28)
1.35 (1.05, 1.74)	0.92 (0.65, 1.30)	**Nivolumab**	0.95 (0.67, 1.33)	0.78 (0.53, 1.16)	0.97 (0.71, 1.34)	0.85 (0.61, 1.18)
1.43 (1.14, 1.79)	0.97 (0.69, 1.35)	1.06 (0.75, 1.48)	**Camrelizumab**	0.83 (0.57, 1.21)	1.03 (0.76, 1.38)	0.90 (0.66, 1.23)
1.72 (1.28, 2.32)	1.17 (0.79, 1.73)	1.27 (0.86, 1.89)	1.21 (0.83, 1.76)	**Toripalimab**	1.24 (0.87, 1.77)	1.09 (0.75, 1.57)
1.39 (1.15, 1.68)	0.94 (0.69, 1.29)	1.03 (0.75, 1.41)	0.97 (0.72, 1.31)	0.81 (0.57, 1.15)	**Pembrolizumab**	0.87 (0.66, 1.17)
1.59 (1.29, 1.96)	1.08 (0.78, 1.49)	1.17 (0.85, 1.63)	1.11 (0.81, 1.52)	0.92 (0.64, 1.33)	1.14 (0.86, 1.52)	**Sintilimab**

Supplementary Table 4 | Individual patient data for chemotherapy (PFS).

No	CheckMate 648		ESCORT-1st		KEYNOTE-590		ASTRUM-007		ORIENT-15		JUPITER-06	
	Time	rate	Time	rate	Time	rate	Time	rate	Time	rate	Time	rate
1	0.00	100.00	0.00	100.00	0.00	100.00	0.00	100.00	0.00	100.00	0.00	100.00
2	0.40	98.49	0.37	99.80	0.26	98.87	0.27	99.42	0.89	98.15	0.14	99.57
3	0.57	97.59	0.64	99.39	0.42	98.02	0.51	99.23	1.09	97.23	0.18	99.14
4	0.69	96.99	0.87	98.79	0.53	97.73	0.72	99.04	1.32	95.84	0.63	98.71
5	0.91	95.78	1.15	97.98	0.69	96.32	0.78	98.27	1.39	93.76	0.69	98.29
6	1.09	94.58	1.24	97.37	0.74	96.03	1.05	97.30	1.46	91.92	1.00	97.86
7	1.26	93.07	1.31	94.94	0.79	94.33	1.20	96.53	1.49	90.76	1.22	97.14
8	1.49	86.45	1.33	93.93	0.90	94.05	1.28	94.99	1.61	90.53	1.26	96.00
9	1.60	84.64	1.35	92.31	0.95	93.77	1.32	93.06	1.71	90.07	1.30	94.43
10	1.89	83.13	1.42	91.30	1.01	92.92	1.39	91.33	1.76	89.38	1.34	92.29
11	2.06	81.63	1.49	90.49	1.06	92.63	1.58	88.44	1.89	88.45	1.39	89.57
12	2.46	79.22	1.56	89.47	1.11	92.35	1.72	87.48	2.06	87.76	1.43	86.71
13	2.63	76.51	1.63	89.07	1.38	91.78	1.91	86.51	2.21	86.84	1.45	85.86
14	2.86	64.76	1.72	88.46	1.54	91.22	1.98	85.74	2.41	86.37	1.57	85.43
15	3.20	63.25	1.93	88.06	1.59	90.93	2.14	84.59	2.53	85.68	1.59	85.00
16	3.55	62.65	2.20	87.45	1.64	90.09	2.29	83.82	2.70	85.22	1.67	84.71
17	3.72	61.75	2.41	87.25	1.80	88.39	2.35	83.04	2.78	83.37	1.79	84.29
18	4.12	60.24	2.55	86.44	1.85	87.82	2.56	82.27	2.83	80.83	2.04	84.00
19	4.35	55.42	2.64	84.62	1.91	86.97	2.65	81.31	2.83	79.68	2.10	83.57
20	5.09	53.31	2.66	83.81	1.96	84.42	2.69	79.38	2.93	78.98	2.12	83.29
21	5.49	51.51	2.73	81.38	2.06	82.44	2.77	76.69	2.95	77.60	2.26	83.00
22	5.78	46.99	2.80	79.35	2.17	80.17	2.82	75.53	2.98	75.98	2.57	82.14
23	5.89	43.98	2.82	78.95	2.22	79.04	2.88	74.95	3.18	75.75	2.61	81.00
24	6.29	42.47	2.89	78.54	2.33	77.90	2.94	73.80	3.33	74.83	2.67	79.71
25	6.58	41.57	3.01	78.34	2.75	77.62	2.99	72.83	3.67	74.36	2.71	78.71
26	6.80	40.06	3.08	77.94	2.96	77.05	3.09	71.87	3.87	73.21	2.75	77.86
27	7.09	36.14	3.19	77.53	3.02	76.49	3.24	70.33	4.00	72.06	2.79	76.43
28	7.32	34.04	3.24	76.92	3.12	76.20	3.70	68.02	4.05	69.75	2.81	76.00
29	7.72	33.43	3.33	76.32	3.18	74.79	3.91	66.09	4.14	68.36	2.93	75.57
30	8.01	32.83	3.42	75.91	3.28	73.94	3.99	64.16	4.19	64.90	3.04	75.14
31	8.29	31.63	3.49	75.51	3.39	73.37	4.02	63.39	4.32	63.97	3.10	74.71
32	8.58	26.81	3.86	75.10	3.44	72.80	4.06	61.27	4.39	61.89	3.26	74.29
33	9.15	25.90	3.93	74.49	3.55	71.67	4.12	58.96	4.62	61.20	3.69	73.86
34	9.55	25.00	3.99	73.48	3.81	71.10	4.16	58.00	4.69	60.05	3.75	73.29
35	9.89	23.19	4.06	73.08	3.92	66.29	4.33	54.34	4.89	59.58	3.85	72.86
36	10.12	20.18	4.11	71.46	4.02	64.02	4.41	53.76	5.11	58.66	3.93	72.43
37	10.92	19.58	4.13	70.45	4.08	61.47	4.46	53.18	5.41	57.74	4.05	70.57
38	11.21	18.07	4.18	69.43	4.18	60.62	4.56	52.22	5.46	55.43	4.07	70.00
39	11.44	17.17	4.25	68.42	4.24	59.77	4.90	51.45	5.53	53.12	4.12	68.14

40	12.01	15.96	4.39	66.60	4.34	58.36	5.13	50.67	5.68	51.04	4.18	67.00
41	12.52	14.16	4.52	65.18	4.45	57.51	5.26	49.52	5.81	49.88	4.26	66.57
42	12.87	12.35	4.61	64.78	4.55	56.37	5.30	48.17	5.83	48.96	4.32	63.29
43	13.55	11.45	4.68	64.37	4.92	55.52	5.46	45.28	6.01	48.04	4.34	60.71
44	13.84	10.84	4.78	63.36	5.03	54.67	5.49	42.97	6.15	47.81	4.46	60.14
45	14.52	9.34	4.87	62.15	5.19	54.39	5.53	39.11	6.45	46.42	4.58	59.57
46	17.38	8.13	5.10	61.34	5.35	52.97	5.59	37.38	6.58	45.73	4.62	58.57
47	19.10	6.33	5.19	60.73	5.45	52.12	5.65	36.22	6.75	44.11	5.11	57.43
48	26.08	4.52	5.23	60.53	5.51	51.56	5.74	35.07	6.85	42.96	5.13	56.86
49			5.33	59.72	5.61	50.71	5.99	33.53	6.90	41.34	5.28	56.29
50			5.40	58.50	5.72	49.58	6.26	32.56	7.02	39.49	5.34	55.71
51			5.44	56.48	5.88	48.73	6.43	31.02	7.10	38.11	5.38	54.71
52			5.46	54.45	5.93	45.04	6.73	30.06	7.25	36.03	5.40	53.57
53			5.49	54.05	5.98	42.78	6.81	27.17	8.02	34.18	5.44	51.86
54			5.53	51.01	6.04	40.51	6.85	26.01	8.21	33.26	5.48	51.14
55			5.58	46.56	6.09	39.09	6.92	23.89	8.29	31.87	5.50	47.57
56			5.67	44.13	6.25	31.73	6.98	22.93	8.39	29.79	5.54	42.57
57			5.72	42.11	6.41	27.20	7.06	22.16	8.51	28.18	5.56	41.43
58			5.79	41.30	7.41	26.91	7.10	21.19	8.64	26.33	5.60	38.14
59			5.83	40.69	7.84	26.35	7.13	20.23	8.78	25.40	5.64	35.71
60			6.04	40.08	7.94	25.78	7.53	19.27	9.60	24.48	5.66	34.43
61			6.20	39.68	8.05	23.51	7.99	18.50	9.73	22.63	5.70	33.71
62			6.38	39.27	8.15	23.23	8.14	17.53	9.83	20.55	5.72	32.43
63			6.47	38.46	8.26	20.40	8.28	15.61	9.93	18.94	5.77	31.57
64			6.64	37.65	8.36	19.26	8.32	13.10	10.27	17.78	5.79	30.86
65			6.73	35.43	8.47	18.13	8.35	12.14	10.87	16.17	6.36	30.14
66			6.75	34.41	9.53	17.85	9.19	10.98	11.27	15.47	6.48	29.43
67			6.82	34.01	9.79	17.28	9.73	10.02	11.71	15.24	6.72	28.57
68			6.84	33.20	10.01	17.00	11.31	9.06	12.23	14.55	6.78	27.86
69			6.89	29.96	10.11	15.01	13.62	7.90	12.88	13.86	6.80	27.14
70			6.91	25.10	10.22	14.16	14.00	6.94	13.28	12.93	6.85	25.43
71			6.96	24.29	10.48	13.03			13.65	12.47	6.95	21.86
72			7.00	23.68	10.69	12.75			13.80	10.85	7.01	21.00
73			7.05	22.87	11.06	12.18			14.05	9.47	7.07	20.29
74			7.07	22.27	11.59	11.61			15.31	8.78	7.11	19.29
75			7.14	21.46	12.23	11.05			16.75	6.70	7.15	18.43
76			7.30	21.05	12.34	9.63					7.50	17.43
77			7.71	20.65	12.60	8.50					7.64	16.57
78			7.99	20.04	14.51	7.65					8.25	15.71
79			8.06	19.64	16.52	6.80					8.29	14.57
80			8.10	18.62	16.68	5.95					8.39	13.57
81			8.13	18.22	17.68	5.38					9.01	10.57
82			8.15	17.81	18.69	4.25					9.64	9.00

83			8.24	16.80	18.85	3.40					9.76	7.57
84			8.33	16.40							9.92	6.00
85			8.43	15.99							12.47	4.00
86			8.52	15.38								
87			8.72	14.98								
88			8.95	14.37								
89			9.57	13.97								
90			9.64	13.16								
91			9.85	12.55								
92			9.87	11.94								
93			9.94	11.54								
94			10.10	10.93								
95			10.40	10.32								
96			10.47	9.92								
97			10.84	9.31								
98			10.95	8.70								
99			11.00	8.10								
100			11.14	7.69								
101			11.18	7.29								
102			11.23	6.48								
103			11.53	5.87								
104			13.84	5.06								
105			14.21	3.85								

Supplementary Table 5 | Individual patient data for chemotherapy (OS).

No	CheckMate 648		ESCORT-1st		KEYNOTE-590		ASTRUM-007		ORIENT-15		JUPITER-06	
	Time	rate	Time	rate	Time	rate	Time	rate	Time	rate	Time	rate
1	0.00	100.00	0.00	100.00	0.00	100.00	0.00	100.00	0.00	100.00	0.00	100.00
2	0.17	99.10	0.41	99.39	0.32	99.06	0.42	99.22	0.40	99.54	0.23	99.30
3	0.46	98.19	0.71	98.99	0.60	98.49	0.86	98.45	0.77	99.31	0.77	98.87
4	0.69	97.29	0.85	98.79	0.78	97.18	1.38	98.06	1.04	98.38	1.04	98.31
5	0.97	96.39	1.15	98.38	0.92	95.67	1.70	97.48	1.64	98.15	1.31	98.17
6	1.14	95.78	1.24	98.18	1.16	94.92	1.84	97.09	1.76	97.69	1.38	97.32
7	1.77	95.18	1.72	97.77	1.76	93.60	1.98	96.31	1.86	97.23	1.85	96.48
8	2.11	93.98	1.79	97.37	2.01	93.22	2.13	95.53	2.01	96.77	2.10	96.06
9	2.51	93.07	1.93	96.96	2.47	92.47	2.31	94.56	2.11	95.84	2.55	95.63
10	2.86	91.57	2.11	96.56	2.71	91.90	2.76	93.79	2.36	95.15	2.78	94.93
11	3.20	90.96	2.18	96.36	2.82	91.15	3.08	93.40	2.51	94.92	3.16	94.51
12	3.43	90.36	2.25	96.15	2.86	90.77	3.13	92.82	2.61	94.23	3.30	94.08
13	3.71	89.46	2.34	95.75	2.96	90.40	3.22	92.23	2.78	92.84	3.48	93.24
14	3.83	87.95	2.41	95.34	3.07	89.83	3.25	91.46	2.90	92.61	3.66	92.96
15	4.17	86.75	2.57	95.14	3.14	89.08	3.69	90.87	3.28	92.15	3.70	92.54
16	4.51	85.24	2.73	94.74	3.21	88.14	3.78	90.10	3.35	91.69	3.75	92.11
17	4.80	84.04	3.07	94.33	3.38	87.19	3.83	89.51	3.62	90.53	3.91	91.83
18	4.97	82.83	3.16	93.93	3.67	86.44	3.97	88.16	3.85	89.84	3.95	91.41
19	5.26	82.23	3.32	93.72	3.74	85.88	4.32	87.18	4.00	89.38	4.00	90.70
20	5.83	76.51	3.49	93.32	3.88	85.12	4.86	84.27	4.07	87.99	4.06	90.14
21	6.29	74.10	3.71	92.92	4.20	84.37	5.04	83.50	4.62	87.30	4.29	89.86
22	6.91	70.48	3.78	92.71	4.30	83.43	5.21	83.11	4.76	86.84	4.33	89.30
23	7.09	68.98	3.81	92.31	4.62	82.86	5.42	81.94	4.89	86.61	4.42	88.87
24	7.49	66.57	4.01	92.11	4.76	82.11	5.49	80.97	5.04	85.91	4.63	87.61
25	7.71	64.76	4.08	91.70	4.87	80.79	5.70	80.00	5.21	84.99	4.72	87.04
26	8.17	62.05	4.10	91.30	4.90	80.41	5.77	78.84	5.31	84.30	4.79	86.76
27	8.51	60.84	4.15	90.89	4.97	80.04	5.95	77.86	5.61	83.60	4.85	86.20
28	8.97	57.83	4.24	90.69	5.01	78.53	6.02	76.89	5.76	82.68	4.97	85.77
29	9.37	55.72	4.52	90.28	5.11	77.02	6.26	76.12	5.88	81.29	5.06	85.35
30	9.77	52.41	4.63	89.88	5.43	76.46	6.33	74.76	6.03	80.37	5.10	84.79
31	10.23	50.30	4.75	89.68	5.78	74.20	6.59	73.98	6.30	77.83	5.46	84.37
32	10.74	49.40	4.93	89.27	6.03	73.45	6.73	72.82	6.50	77.14	5.71	83.94
33	11.14	46.99	5.09	89.07	6.17	73.07	7.12	71.65	6.55	75.98	5.87	83.38
34	11.43	46.08	5.18	88.87	6.35	72.69	7.52	70.68	6.73	75.06	6.19	82.96
35	12.00	43.98	5.23	88.26	6.49	71.56	7.71	69.32	6.87	73.67	6.30	82.25
36	12.34	42.17	5.34	88.06	6.56	70.81	7.99	68.74	7.00	72.52	6.37	81.27
37	12.57	41.57	5.53	87.85	6.59	70.24	8.15	67.38	7.40	71.36	6.59	80.85
38	12.74	41.27	5.62	86.84	6.63	69.68	8.24	65.83	7.64	70.44	6.86	79.58
39	12.91	40.06	5.71	86.03	6.66	69.11	8.36	64.66	7.94	69.75	6.98	79.01

40	13.31	38.86	5.85	85.63	6.73	67.98	8.50	64.27	8.14	69.05	7.07	78.31
41	13.66	37.95	5.96	85.43	6.81	67.42	8.71	61.94	8.46	66.74	7.11	77.75
42	13.94	36.75	6.05	85.02	6.95	67.23	9.01	60.19	8.64	65.59	7.18	77.18
43	14.51	35.54	6.12	84.41	7.19	66.86	9.18	58.25	8.76	65.13	7.20	75.92
44	15.83	32.53	6.26	83.60	7.26	66.29	9.69	56.70	8.96	64.20	7.31	75.21
45	17.37	28.61	6.44	83.00	7.40	64.60	10.18	55.73	9.18	63.51	7.34	74.79
46	18.29	25.60	6.49	82.59	7.51	63.65	10.30	54.37	9.36	63.05	7.45	72.68
47	18.86	25.30	6.58	82.19	7.65	62.71	10.41	53.79	9.43	62.36	7.59	72.11
48	19.54	24.70	6.65	80.57	7.76	62.15	10.53	53.01	9.53	61.20	7.63	71.41
49	20.74	23.80	6.79	79.76	7.86	61.39	10.63	52.04	9.78	60.28	7.95	70.70
50	21.03	22.89	7.02	79.35	8.00	59.70	11.40	51.26	9.90	58.89	8.04	70.14
51	22.23	21.99	7.06	78.54	8.11	58.57	11.84	49.32	10.22	58.43	8.08	69.30
52	23.31	21.08	7.18	78.14	8.39	57.06	12.19	48.54	10.35	56.81	8.17	68.45
53	24.00	18.67	7.27	77.73	8.57	55.93	12.40	47.77	10.57	56.35	8.24	67.75
54	25.94	17.47	7.50	77.13	8.78	54.99	13.03	47.18	11.07	55.43	8.60	66.90
55	26.57	16.27	7.57	76.52	8.89	53.67	13.78	45.24	11.17	54.50	9.03	66.06
56	28.11	14.16	7.68	75.71	9.06	53.11	13.85	43.50	11.37	52.89	9.08	65.35
57			7.73	75.30	9.13	52.35	13.94	42.72	11.69	52.19	9.21	64.51
58			7.80	74.70	9.34	51.98	13.99	40.97	12.11	50.81	9.26	63.38
59			7.93	73.89	9.45	51.60	14.13	40.00	12.36	50.12	9.48	62.54
60			7.96	73.48	9.59	50.47	14.62	38.84	12.61	49.42	9.62	61.69
61			8.05	72.87	9.73	50.09	15.11	37.67	12.71	48.27	9.64	60.70
62			8.14	72.67	9.77	48.96	15.74	36.50	12.83	47.58	9.93	59.58
63			8.28	71.66	9.98	48.78	15.90	34.95	13.33	46.65	10.14	58.59
64			8.32	71.46	10.12	48.40	16.14	33.79	13.72	45.96	10.43	57.46
65			8.53	71.05	10.44	47.65	17.61	32.23	13.87	45.27	10.50	56.48
66			8.62	70.65	10.54	47.08	17.72	30.87	14.47	44.57	10.52	54.37
67			8.67	70.45	10.61	46.14	18.19	28.93	14.64	43.42	10.77	53.24
68			8.71	69.23	10.72	45.39	18.89	27.18	14.79	42.26	10.79	52.11
69			8.78	68.83	10.82	44.63	19.05	25.44	15.06	41.57	10.93	50.99
70			8.85	68.42	11.00	44.44	22.37	22.52	15.19	40.65	11.02	49.86
71			8.87	67.81	11.14	43.31	22.46	19.81	15.66	39.72	11.08	48.73
72			9.10	67.41	11.21	42.18	24.92	14.76	15.86	38.34	11.42	47.61
73			9.19	66.80	11.39	41.81	25.13	10.10	16.40	38.11	11.56	43.66
74			9.26	65.79	11.71	40.49			16.55	36.72	12.37	42.25
75			9.31	65.59	11.78	39.36			16.78	36.03	12.53	39.72
76			9.56	64.78	11.85	38.61			16.85	35.33	12.80	38.31
77			9.68	63.97	11.95	37.85			17.30	34.18	13.03	37.18
78			9.79	63.16	12.09	36.91			17.99	33.49	13.07	35.77
79			9.91	61.94	12.41	36.53			18.09	32.33	13.09	34.37
80			10.02	61.13	12.59	35.40			18.29	31.41	13.39	32.96
81			10.16	60.12	12.83	35.03			18.49	30.49	14.38	31.27
82			10.20	59.11	12.94	34.28			18.76	29.56	15.24	29.44

83			10.39	57.89	13.47	33.52			19.51	28.41	15.40	27.61
84			10.69	57.09	13.65	33.15			19.73	27.48	16.30	25.63
85			10.78	56.28	13.82	32.96			20.25	26.33	18.29	23.24
86			10.98	55.47	13.89	32.02			20.99	24.94	18.51	20.85
87			11.21	54.86	14.07	31.64			21.12	23.33	19.28	17.46
88			11.28	53.64	14.24	31.26			21.76	21.94		
89			11.42	52.63	14.42	30.89			22.14	20.09		
90			11.56	50.20	14.49	30.32			23.13	17.78		
91			11.88	49.60	14.56	29.38			23.95	15.70		
92			12.06	49.19	14.63	29.00						
93			12.29	48.79	14.77	28.63						
94			12.45	48.38	14.91	28.25						
95			12.63	47.98	15.16	27.50						
96			12.73	46.96	15.34	27.12						
97			12.79	46.15	15.41	26.74						
98			12.86	45.75	15.58	25.99						
99			13.02	45.34	16.01	25.61						
100			13.09	44.33	16.47	25.05						
101			13.25	43.72	16.57	24.86						
102			13.30	42.31	16.71	24.11						
103			13.53	41.70	17.24	23.54						
104			13.76	41.30	17.56	23.16						
105			13.96	40.69	17.88	22.79						
106			14.31	40.28	18.37	22.03						
107			14.58	39.07	18.58	21.66						
108			14.74	37.65	18.83	21.09						
109			14.88	37.04	18.97	20.72						
110			15.09	36.44	19.39	20.34						
111			15.36	35.63	19.53	19.96						
112			15.64	34.82	20.13	19.21						
113			15.75	34.01	20.34	18.83						
114			16.23	32.39	21.44	18.27						
115			16.76	31.38	22.00	17.70						
116			16.85	30.57	22.07	17.14						
117			17.31	29.55	22.11	16.95						
118					30.18	12.62						
119					32.23	6.21						

Supplementary Table 6 | Summary of statistical goodness-of-fit of Kaplan-Meier curves for chemotherapy.

	Exponential	Weibull	Log-logistic	Log-normal	Gompertz
**CheckMate 648-PFS**					
parameter 1	0.09675670	0.05603880	1.98218400	1.95729400	0.09036670
parameter 2	-	0.18565460	-0.63455540	-0.08060890	0.00760450
AIC	907.016400	892.061900	875.133300	871.508600	907.718400
BIC	910.797100	899.623400	882.694800	879.070100	915.279900
**CheckMate 648-OS**					
parameter 1	0.04685740	0.01763000	2.73783500	2.69611300	0.03539420
parameter 2	-	0.26621130	-0.61988540	0.01734780	0.01921040
AIC	900.442200	875.947600	875.217000	906.631600	889.735500
BIC	904.222900	883.509000	882.778500	914.193100	897.296900
**ESCORT-1st-PFS**					
parameter 1	0.10560060	0.01904830	2.05509700	2.01355000	0.05833620
parameter 2	-	0.53355410	-0.94638490	-0.34591540	0.08600940
AIC	741.402900	644.890600	637.233400	649.431300	687.392400
BIC	745.100000	652.284800	644.627600	656.825500	694.786600
**ESCORT-1st-OS**					
parameter 1	0.04031490	0.00453990	2.88160500	2.87085700	0.01723840
parameter 2	-	0.54763450	-0.78043360	-0.14525520	0.07171260
AIC	724.966000	663.177400	665.432000	685.941100	675.189800
BIC	728.663100	670.571600	672.826200	693.335300	682.584000
**KEYNOTE-590-PFS**					
parameter 1	0.11053250	0.04103180	1.95662400	1.89683200	0.08167250
parameter 2	-	0.33468420	-0.75786630	-0.15039160	0.04046700
AIC	723.990700	683.668300	686.991700	699.571200	707.550300
BIC	727.603900	690.894500	694.217900	706.797500	714.776500
**KEYNOTE-590-OS**					
parameter 1	0.05939110	0.01873320	2.56754400	2.49872600	0.04129220
parameter 2	-	0.31174530	-0.71984460	-0.09321170	0.02456700
AIC	728.883300	692.877400	712.004500	730.335200	705.780900
BIC	732.496500	700.103600	719.230800	737.561500	713.007100

**ASTRUM-007-PFS**					
parameter 1	0.11415220	0.02976800	1.93444800	1.91372800	0.07625760
parameter 2	-	0.45033640	-0.90052060	-0.31515620	0.06325680
AIC	466.029700	422.728600	405.615200	411.858700	450.152200
BIC	469.239200	429.147600	412.034100	418.277600	456.571100
**ASTRUM-007-OS**					
parameter 1	0.04265060	0.00768000	2.87787400	2.84291600	0.02259790
parameter 2	-	0.42383320	-0.71668990	-0.14778560	0.04092910
AIC	477.929400	446.002500	453.480600	457.265900	451.934500
BIC	481.138900	452.421400	459.899500	463.684900	458.353500
**ORIENT-15-PFS**					
parameter 1	0.09504260	0.02568620	2.10214200	2.07818200	0.06224160
parameter 2	-	0.41099730	-0.81732440	-0.26383630	0.05329040
AIC	855.939000	788.291600	777.124600	774.155500	823.957300
BIC	859.744200	795.901900	784.734800	781.765800	831.567600
**ORIENT-15-OS**					
parameter 1	0.04470090	0.00839040	2.83287800	2.79202000	0.02378480
parameter 2	-	0.42212310	-0.70888150	-0.12817700	0.04347640
AIC	868.656000	810.871700	825.168100	835.701300	821.463700
BIC	872.461100	818.482000	832.778400	843.311600	829.073900
**JUPITER-06-PFS**					
parameter 1	0.11979050	0.02086470	1.95548700	1.88918200	0.05786710
parameter 2	-	0.56311860	-0.91762570	-0.29418950	0.11725410
AIC	638.708300	548.206100	565.481800	584.106600	573.195000
BIC	642.257300	555.304300	572.580000	591.204700	580.293200
**JUPITER-06-OS**					
parameter 1	0.04753550	0.00377300	2.80157100	2.76775800	0.01839160
parameter 2	-	0.60823730	-0.89564670	-0.22319170	0.07567190
AIC	636.186300	555.021800	563.267200	597.242800	567.152900
BIC	639.735400	562.120000	570.365300	604.340900	574.251000

AIC, Akaike information criterion; BIC, Bayesian Information Criterion; PFS, progression-free survival; OS, overall survival.

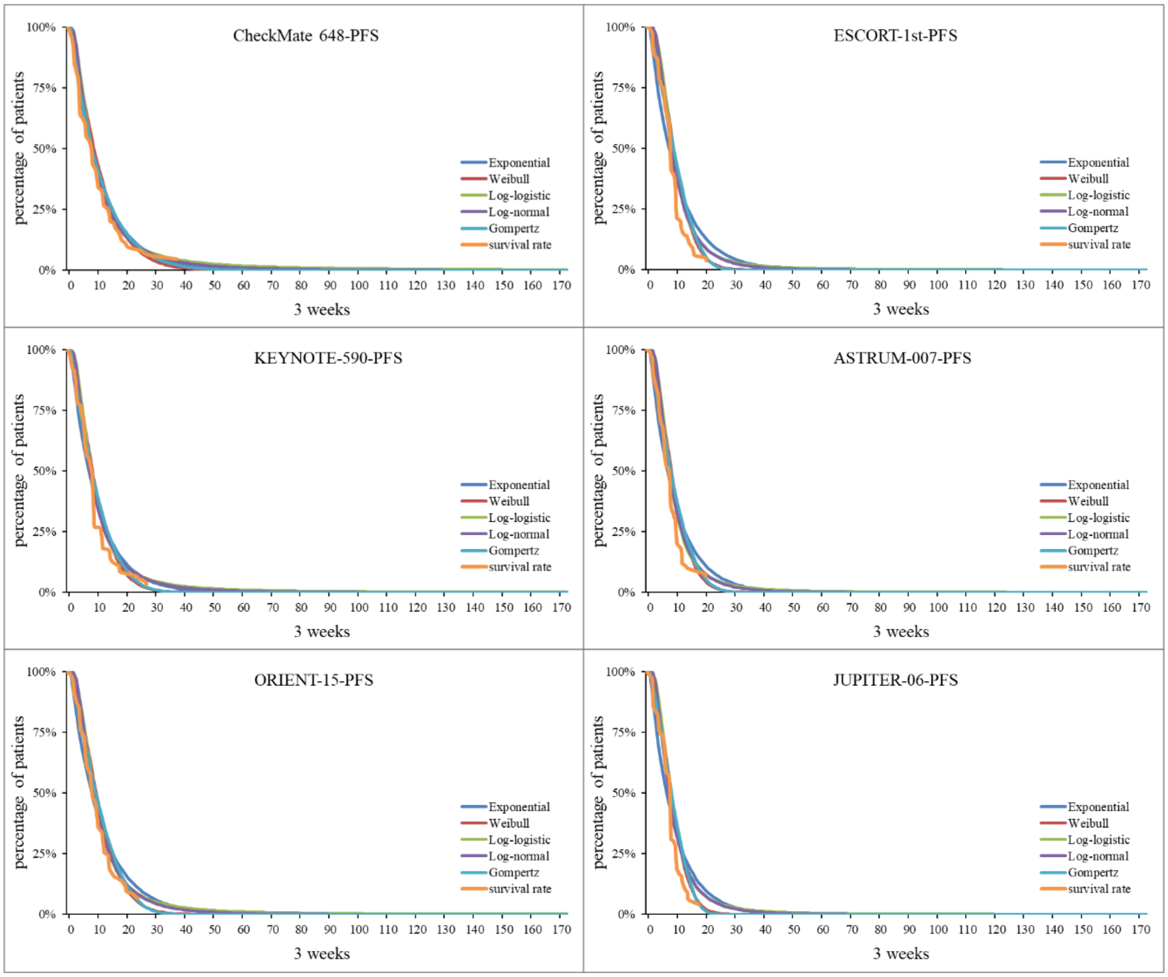

Supplementary Figure 3 | The reconstructed Kaplan-Meier PFS curves of chemotherapy.

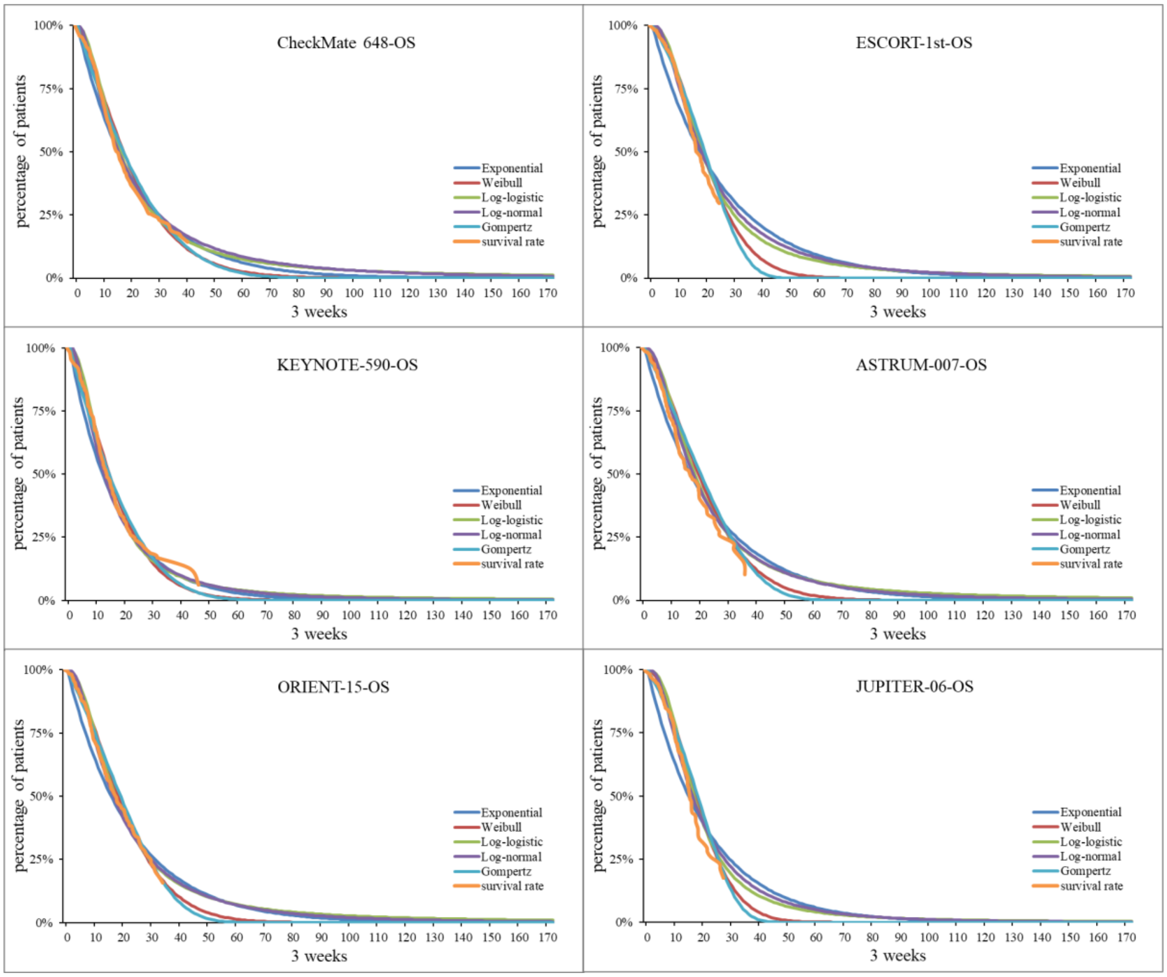

Supplementary Figure 4 | The reconstructed Kaplan-Meier OS curves of chemotherapy.

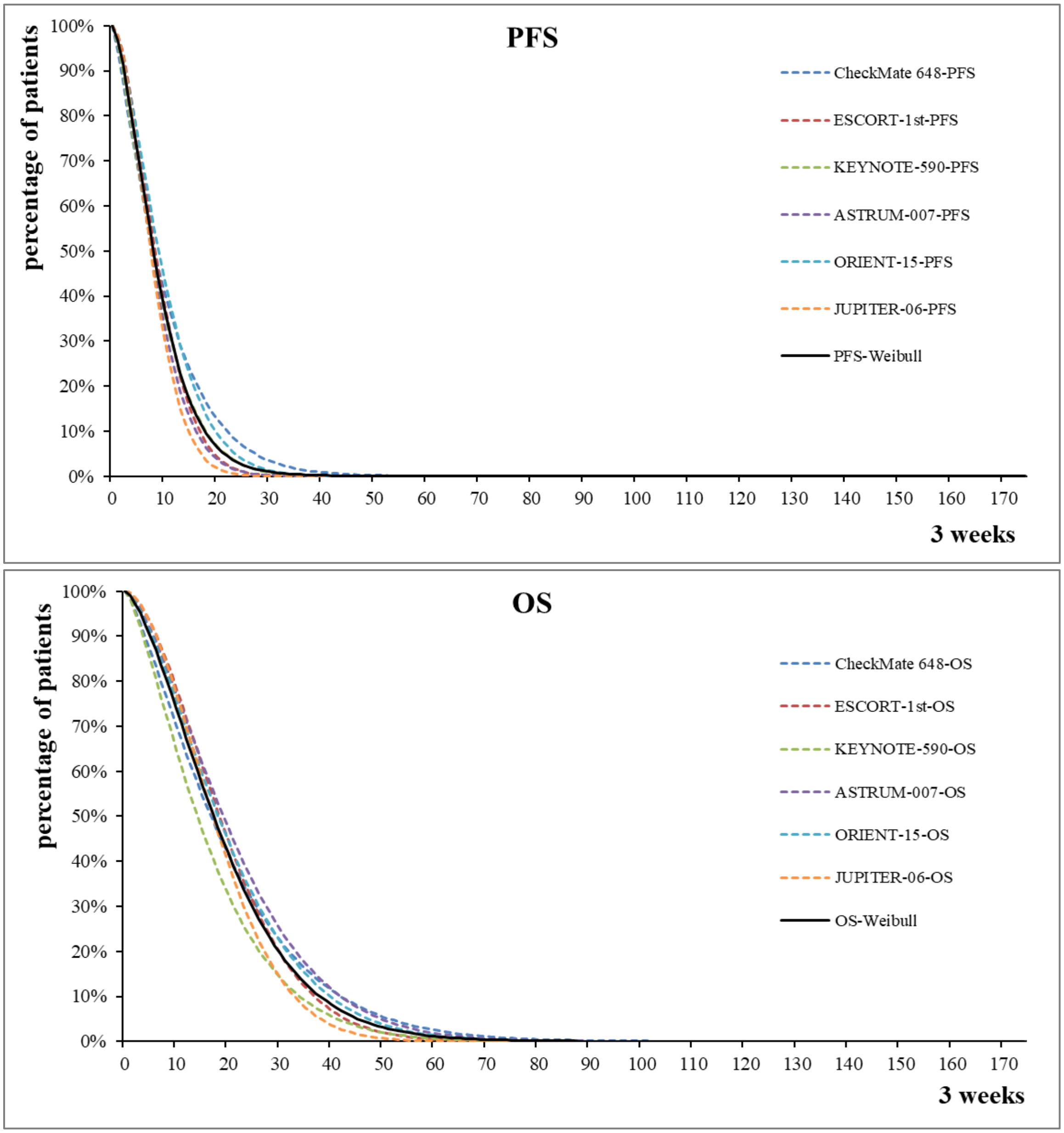

Supplementary Figure 5 | PFS and OS curves for the chemotherapy group fitted by Weibull distribution.

Supplementary Table 7 | Scenario analysis results for different sources of utility values.

Strategy	Total cost	QALYs	ICER ($/QALY, pairwise comparison)					
**Utility values--Zhang PF et al.**								
Chemotherapy	4,436.40	0.67	-	-	-	-	-	-
Toripalimab plus chemotherapy	8,110.53	0.93	14,326.47	-	-	-	-	-
Sintilimab plus chemotherapy	8,643.48	0.89	18,929.29	dominated	-	-	-	-
Camrelizumab plus chemotherapy	9,656.62	0.84	30,140.04	dominated	dominated	-	-	-
Serplulimab plus chemotherapy	36,370.68	0.85	173,603.19	dominated	dominated	2,484,784.03	-	-
Pembrolizumab plus chemotherapy	37,312.48	0.82	214,763.41	dominated	dominated	dominated	dominated	-
Nivolumab plus chemotherapy	56,972.21	0.80	410,210.50	dominated	dominated	dominated	dominated	dominated
**Utility values--Zhang QL et al.**								
Chemotherapy	4,436.40	0.56	-	-	-	-	-	-
Toripalimab plus chemotherapy	8,110.53	0.77	16,956.43	-	-	-	-	-
Sintilimab plus chemotherapy	8,643.48	0.75	21,590.81	dominated	-	-	-	-
Camrelizumab plus chemotherapy	9,656.62	0.72	32,902.23	dominated	dominated	-	-	-
Serplulimab plus chemotherapy	36,370.68	0.72	196,492.54	dominated	dominated	6,915,188.07	-	-
Pembrolizumab plus chemotherapy	37,312.48	0.69	244,100.40	dominated	dominated	dominated	dominated	-
Nivolumab plus chemotherapy	56,972.21	0.66	506,861.37	dominated	dominated	dominated	dominated	dominated
**Utility values--Marguet S et al.**								
Chemotherapy	4,436.40	0.72	-	-	-	-	-	-
Toripalimab plus chemotherapy	8,110.53	0.99	13,459.33	-	-	-	-	-
Sintilimab plus chemotherapy	8,643.48	0.95	18,173.48	dominated	-	-	-	-

Camrelizumab plus chemotherapy	9,656.62	0.90	29,772.90	dominated	dominated	-	-	-
Serplulimab plus chemotherapy	36,370.68	0.91	167,423.46	dominated	dominated	1,734,114.88	-	-
Pembrolizumab plus chemotherapy	37,312.48	0.88	206,609.65	dominated	dominated	dominated	dominated	-
Nivolumab plus chemotherapy	56,972.21	0.86	376,020.60	dominated	dominated	dominated	dominated	dominated
QALYs, quality adjusted life-years; ICER, incremental cost-effectiveness ratios.								

Supplementary Table 8 | Scenario analysis results of various time horizons.

Strategy	Total cost	QALYs	ICER ($/QALY, pairwise comparison)					
**Time horizon = 2 years**								
Chemotherapy	4,216.75	0.65	-	-	-	-	-	-
Toripalimab plus chemotherapy	7,239.05	0.82	17,977.48	-	-	-	-	-
Sintilimab plus chemotherapy	7,886.06	0.80	24,197.60	dominated	-	-	-	-
Camrelizumab plus chemotherapy	8,999.18	0.78	38,939.39	dominated	dominated	-	-	-
Serplulimab plus chemotherapy	35,144.39	0.78	239,102.45	dominated	dominated	4,002,879.16	-	-
Pembrolizumab plus chemotherapy	36,299.16	0.76	292,875.16	dominated	dominated	dominated	dominated	-
Nivolumab plus chemotherapy	56,167.02	0.74	571,170.92	dominated	dominated	dominated	dominated	dominated
**Time horizon = 5 years**								
Chemotherapy	4,435.70	0.69	-	-	-	-	-	-
Toripalimab plus chemotherapy	8,086.06	0.94	14,147.08	-	-	-	-	-
Sintilimab plus chemotherapy	8,627.46	0.91	18,741.00	dominated	-	-	-	-
Camrelizumab plus chemotherapy	9,647.45	0.86	29,925.92	dominated	dominated	-	-	-
Serplulimab plus chemotherapy	36,357.94	0.87	172,156.63	dominated	dominated	2,369,947.74	-	-
Pembrolizumab plus chemotherapy	37,303.94	0.84	212,635.51	dominated	dominated	dominated	dominated	-
Nivolumab plus chemotherapy	56,965.88	0.82	403,200.93	dominated	dominated	dominated	dominated	dominated
**Time horizon = 8 years**								
Chemotherapy	4,436.40	0.69	-	-	-	-	-	-
Toripalimab plus chemotherapy	8,109.98	0.95	14,049.81	-	-	-	-	-
Sintilimab plus chemotherapy	8,643.21	0.91	18,624.47	dominated	-	-	-	-

Camrelizumab plus chemotherapy	9,656.51	0.86	29,773.14	dominated	dominated	-	-	-
Serplulimab plus chemotherapy	36,370.54	0.87	170,928.87	dominated	dominated	2,323,488.34	-	-
Pembrolizumab plus chemotherapy	37,312.40	0.84	211,365.28	dominated	dominated	dominated	dominated	-
Nivolumab plus chemotherapy	56,972.15	0.82	400,794.51	dominated	dominated	dominated	dominated	dominated
**Time horizon = 10 years**								
Chemotherapy	4,436.40	0.69	-	-	-	-	-	-
Toripalimab plus chemotherapy	8,110.53	0.95	14,047.53	-	-	-	-	-
Sintilimab plus chemotherapy	8,643.48	0.91	18,622.34	dominated	-	-	-	-
Camrelizumab plus chemotherapy	9,656.62	0.86	29,771.17	dominated	dominated	-	-	-
Serplulimab plus chemotherapy	36,370.68	0.87	170,911.36	dominated	dominated	2,322,505.88	-	-
Pembrolizumab plus chemotherapy	37,312.48	0.84	211,350.41	dominated	dominated	dominated	dominated	-
Nivolumab plus chemotherapy	56,972.21	0.82	400,768.95	dominated	dominated	dominated	dominated	dominated
QALYs, quality adjusted life-years; ICER, incremental cost-effectiveness ratios.								

Supplementary Table 9 | Scenario analysis results related to lower and higher patient weight and body surface area.

Strategy	Total cost	QALYs	ICER ($/QALY, pairwise comparison)					
**Lower patient weight and body surface area**								
Chemotherapy	4,321.06	0.69	-	-	-	-	-	-
Toripalimab plus chemotherapy	8,064.09	0.95	14,310.94	-	-	-	-	-
Sintilimab plus chemotherapy	8,643.48	0.91	19,132.86	dominated	-	-	-	-
Camrelizumab plus chemotherapy	9,610.02	0.86	30,163.17	dominated	dominated	-	-	-
Serplulimab plus chemotherapy	32,851.75	0.87	152,695.41	dominated	dominated	2,020,623.31	-	-
Pembrolizumab plus chemotherapy	37,219.34	0.84	211,493.09	dominated	dominated	dominated	dominated	-
Nivolumab plus chemotherapy	56,904.21	0.82	401,130.07	dominated	dominated	dominated	dominated	dominated
**Higher patient weight and body surface area**								
Chemotherapy	4,686.29	0.69	-	-	-	-	-	-
Toripalimab plus chemotherapy	8,211.15	0.95	13,476.81	-	-	-	-	-
Sintilimab plus chemotherapy	8,643.48	0.91	17,516.22	dominated	-	-	-	-
Camrelizumab plus chemotherapy	9,757.58	0.86	28,921.84	dominated	dominated	-	-	-
Pembrolizumab plus chemotherapy	37,514.29	0.84	211,041.28	dominated	dominated	dominated	-	-
Serplulimab plus chemotherapy	43,913.73	0.87	209,944.17	dominated	dominated	2,969,517.10	204,490.89	-
Nivolumab plus chemotherapy	57,119.53	0.82	399,986.54	dominated	dominated	dominated	dominated	dominated
QALYs, quality adjusted life-years; ICER, incremental cost-effectiveness ratios.								

Supplementary Table 10 | Scenario analysis results related to subsequent treatment.

A. Subsequent treatment and patient proportion.

	treatment strategy	Cost	Cost per cycle ($)	Chemotherapy group	Chemoimmunotherapy group
Camrelizumab	200mg / 2-week	2928.00 CNY/ 200mg	693.37	20%	10%
Tislelizumab	200mg / 3-week	1450.00 CNY/ 100mg	457.82		
Anlotinib	12mg / day, 2-week treatment, 1 week discontinuation	1785.00 CNY/ 70mg	676.32	10%	10%
Apatinib	850mg / day	1571.40 CNY/ 4250mg	1041.93		
Docetaxel	75 mg / m² / 3-week	22.60 CNY/ 20mg	23.01	25%	20%
Best supportive care	3-week	182.23$	182.23	45%	60%

B. Scenario analysis results related to subsequent treatment.

Strategy	Total cost	QALYs	ICER ($/QALY, pairwise comparison)					
Chemotherapy	4,436.40	0.72	-	-	-	-	-	-
Toripalimab plus chemotherapy	8,110.53	0.99	13,459.33	-	-	-	-	-
Sintilimab plus chemotherapy	8,643.48	0.95	18,173.48	dominated	-	-	-	-
Camrelizumab plus chemotherapy	9,656.62	0.90	29,772.90	dominated	dominated	-	-	-
Serplulimab plus chemotherapy	36,370.68	0.91	167,423.46	dominated	dominated	1,734,114.88	-	-
Pembrolizumab plus chemotherapy	37,312.48	0.88	206,609.65	dominated	dominated	dominated	dominated	-
Nivolumab plus chemotherapy	56,972.21	0.86	376,020.60	dominated	dominated	dominated	dominated	dominated
QALYs, quality adjusted life-years; ICER, incremental cost-effectiveness ratios.								

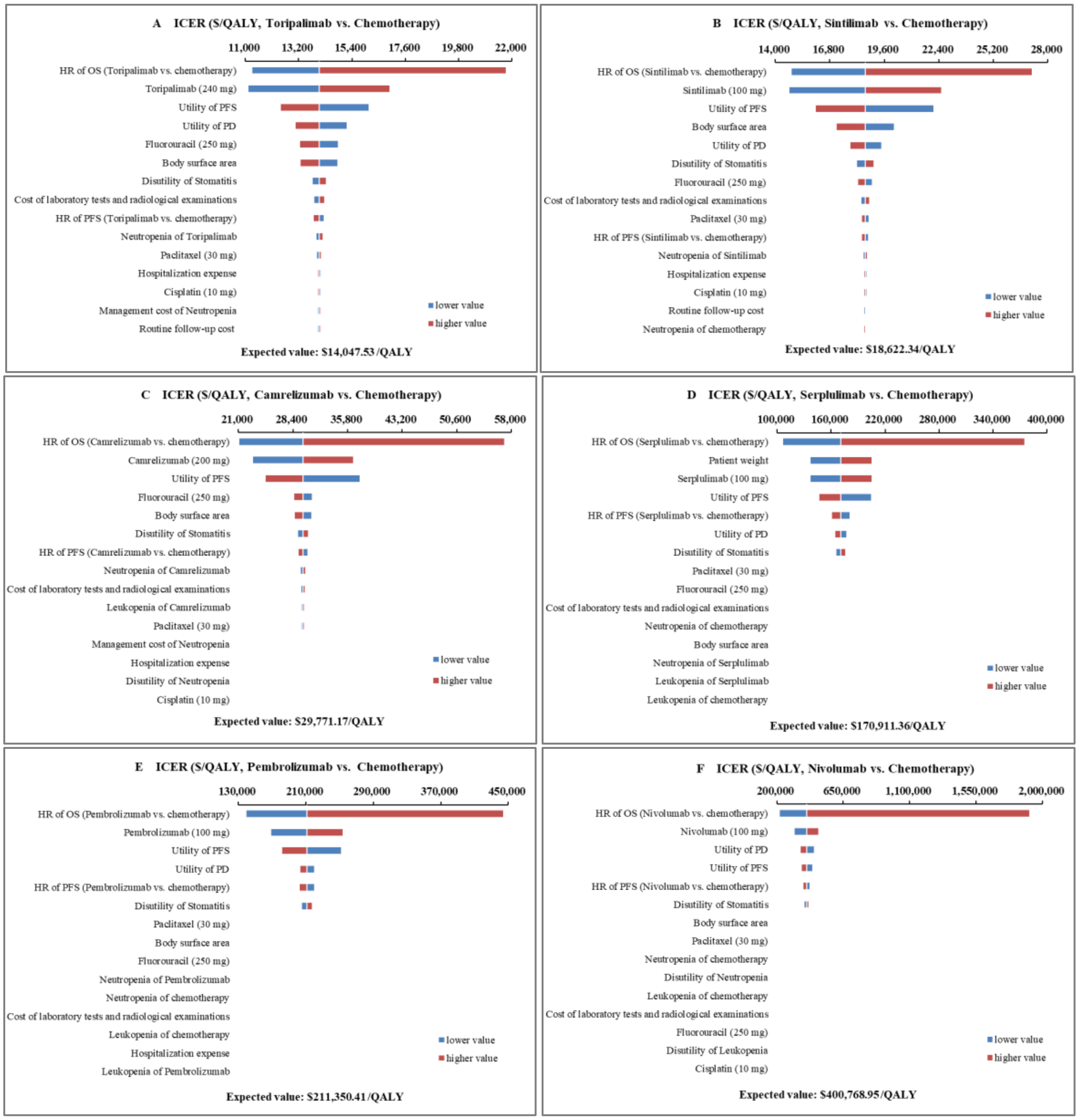

Supplementary Figure 6 | Tornado diagrams of one-way sensitivity analyses. (A: Toripalimab vs. Chemotherapy; B: Sintilimab vs. Chemotherapy; C: Camrelizumab vs. Chemotherapy; D: Serplulimab vs. Chemotherapy; E: Pembrolizumab vs. Chemotherapy; F: Nivolumab vs. Chemotherapy)
